# N6-Methyladenosine RNA Methylation Regulator-Related Alternative Splicing (AS) Gene Signature Predicts Non–Small Cell Lung Cancer Prognosis

**DOI:** 10.3389/fmolb.2021.657087

**Published:** 2021-06-11

**Authors:** Zhenyu Zhao, Qidong Cai, Pengfei Zhang, Boxue He, Xiong Peng, Guangxu Tu, Weilin Peng, Li Wang, Fenglei Yu, Xiang Wang

**Affiliations:** ^1^ Department of Thoracic Surgery, The Second Xiangya Hospital of Central South University, Changsha, China; ^2^ Hunan Key Laboratory of Early Diagnosis and Precise Treatment of Lung Cancer, The Second Xiangya Hospital of Central South University, Changsha, China

**Keywords:** non–small cell lung cancer, m6A, alternative splicing, The Cancer Genome Atlas, prognostic signature

## Abstract

Aberrant N6-methyladenosine (m6A) RNA methylation regulatory genes and related gene alternative splicing (AS) could be used to predict the prognosis of non–small cell lung carcinoma. This study focused on 13 m6A regulatory genes (METTL3, METTL14, WTAP, KIAA1429, RBM15, ZC3H13, YTHDC1, YTHDC2, YTHDF1, YTHDF2, HNRNPC, FTO, and ALKBH5) and expression profiles in TCGA-LUAD (*n* = 504) and TCGA-LUSC (*n* = 479) datasets from the Cancer Genome Atlas database. The data were downloaded and bioinformatically and statistically analyzed, including the gene ontology and Kyoto Encyclopedia of Genes and Genomes pathway enrichment analyses. There were 43,948 mRNA splicing events in lung adenocarcinoma (LUAD) and 46,020 in lung squamous cell carcinoma (LUSC), and the data suggested that m6A regulators could regulate mRNA splicing. Differential HNRNPC and RBM15 expression was associated with overall survival (OS) of LUAD and HNRNPC and METTL3 expression with the OS of LUSC patients. Furthermore, the non–small cell lung cancer prognosis-related AS events signature was constructed and divided patients into high- *vs.* low-risk groups using seven and 14 AS genes in LUAD and LUSC, respectively. The LUAD risk signature was associated with gender and T, N, and TNM stages, but the LUSC risk signature was not associated with any clinical features. In addition, the risk signature and TNM stage were independent prognostic predictors in LUAD and the risk signature and T stage were independent prognostic predictors in LUSC after the multivariate Cox regression and receiver operating characteristic analyses. In conclusion, this study revealed the AS prognostic signature in the prediction of LUAD and LUSC prognosis.

## Introduction

Lung cancer is still the most significant health burden in the world, accounting for 14% of all newly diagnosed cancer cases as the second most common cancer and 18% of all cancer-related deaths as the leading cause of cancer death globally in 2018 and 2020 (de Martel et al., 2020; Sung et al., 2021). Lung cancer is also prevalent and the leading cause of cancer death in men (Sung et al., 2021). Histologically, lung cancer can be divided into small cell lung cancer and non–small cell lung cancer (NSCLC), and the latter accounts for 85% of all lung cancer cases, and the overall 5-year survival rate of lung cancer remains to be approximately 15% (Balata et al., 2019). NSCLC can be further classified as lung squamous cell carcinoma (LUSC), lung adenocarcinoma (LUAD), and larger cell carcinoma; however, LUAD and LUSC are the main histological subtypes of NSCLC (Tanoue et al., 2015) and major contributors to NSCLC morbidity and mortality (Hirsch et al., 2017). The outcome data were from our most recent advancement and improvement in early detection, prevention, improved surgical procedures, neoadjuvant therapy, immunotherapy, and targeted therapy. To date, treatment of NSCLC is dependent on the stage of disease at diagnosis, and early-staged NSCLC could be surgically cured, whereas the advanced staged diseases can only be subjected to chemotherapy, radiation therapy, immunotherapy, and/or targeted therapy (Maconachie et al., 2019; Planchard et al., 2018) and their prognosis is, therefore, still poor, approximately less than 5–7% at the best according to the American Cancer Society data (https://www.cancer.org/cancer/lung-cancer/detection-diagnosis-staging/survival-rates.html) or after advanced therapy (Zhang et al., 2021). Thus, the search and development of biomarkers for early detection and prediction of prognosis and treatment outcome are urgently needed to effectively conquer this now deadly disease clinically.

Newly transcribed RNA could undergo different chemical modifications and N6-methyladenosine (m6A) is the most prevailing one in polyadenylated RNAs (Bokar et al., 1997). Methylation of the adenosine is directed in cells by a large m6A methyltransferase complex containing METTL3 as the SAM-binding subunit (Bokar et al., 1997). The biological functions of m6A are through a group of RNA-binding proteins that can specifically recognize the methylated adenosine on RNA molecules to regulate cell activities (Ji et al., 2018), for example, N6-methyladenosine (m6A) RNA modification could regulate RNA splicing, stability, translocation, and translation and therefore, to influence gene expression and functions in cells (Deng, et al., 2018). These binding proteins to m6A are regarded as the m6A readers, and m6A methyltransferases are considered as the writers, whereas demethylases are considered as the erasers. Altogether, these proteins form a complex mechanism of m6A regulation in which writers and erasers determine the distributions of m6A on RNA, whereas readers mediate m6A-dependent functions (Liu et al., 2014; Wang et al., 2014). Deregulation of the m6A on an RNA molecule has been implicated in the development of various human cancers (Liu et al., 2014; Wang et al., 2014). According to the recent studies, there were 13 m6A regulator genes confirmed to affect cancer progression, including the “writer” (KIAA1429, METTL3, METTL14, RBM15, WTAP, and ZC3H13), the “readers” (HNRNPC, YTHDC1, YTHDC2, YTHDF1, and YTHDF2), and the “erasers” (ALKBH5 and FTO) (Zhang et al., 2020c; Zhang et al., 2020b). Further studies of the m6A regulator genes showed that the m6A regulator genes were also the mRNA splicing factors for gene alternative splicing (GAS) and the m6A regulator genes could interact with the AS events (Kasowitz et al., 2018; Yoshimi et al., 2019). Human cancer cells frequently showed the GAS events, which were regulated by the m6A regulators (Dai et al., 2018). For example, METTL3 was able to regulate the mRNA alternative splicing by the p53 pathway (Alarcón et al., 2015). YTHDC1 could recruit SRSF10 to its target mRNA regions and modulate their exon skipping (Xiao et al., 2016). Abnormal splicing factor expression in normal cells could lead to the formation of the specific pro-oncogenic splicing subtypes and carcinogenesis (Kasowitz et al., 2018).

Indeed, gene alternative splicing (GAS), a posttranscriptional process, subjects a single pre-mRNA molecule to splice into different exons for coding and expression of various protein isoforms (David and Manley, 2008). A molecular structure called a spliceosome is assembled on the pre-mRNA to join the exons together at the splicing site to form a particular mRNA molecule, while the introns are discarded (Papasaikas and Valcárcel, 2016). The assembly of spliceosomes on pre-mRNA is usually affected by the SF and some exons (alternative exons) are variably incorporated into mRNA; thus, under different alternative splicing patterns (including exon skip, retained intron, alternate donor site, alternate acceptor site, alternate promoter, alternate terminator, and mutually exclusive exons), the whole exons of a gene could be spliced into mRNA or excluded (Hanahan and Weinberg, 2011). The different GAS events could lead to the diversity of protein functions and normal GAS will maintain normal cell functions, which is mediated by the production of the diverse and multifunctional proteome to ensure “normal” RNA molecules to maintain normal cell functions; however, abnormal GAS will promote tumorigenesis and cancer development (Bonnal et al., 2020), which could be mainly due to the up or downregulation of the related splicing factors, for example, alterations in the upstream signaling pathways or mutations in the splicing site sequences all lead to abnormal mRNA splicing (Li et al., 2019). Accumulating evidence suggests the contribution of abnormal GAS to cancer phenotypes, like increases in cell proliferation, angiogenesis, but inhibition of apoptosis and drug resistance, and the GAS events form a novel and separate hallmark in cancer (Bonnal et al., 2020; Urbanski et al., 2018). For example, in gastric cancer, abnormal GAS could lead to activation of tumor cell invasion and metastasis (Pio and Montuenga, 2009; Sun and Ma, 2019), while in breast cancer, abnormal GAS results in drug resistance (Yang et al., 2019). In lung cancer, the GAS events could be used as biomarkers for tumor diagnosis (Sholl, 2017). Aberrant *BCL2L1*, *MDM2*, *MDM4*, *NUMB*, and *MET* mRNA splicing occurred in lung cancer and altered cell apoptosis, proliferation, and cohesion (Coomer, Black). Thus, further investigation related to the abnormal GAS events to lung tumorigenesis (Coomer et al., 2019) and novel strategy for cancer targeting therapy (Frankiw et al., 2019) as well as biomarkers for various human cancers, including NSCLC (Li et al., 2017; Paschalis et al., 2018; Yang et al., 2019; Liu et al., 2020; Zhang et al., 2020a).

In this study, we focused on the m6a-related splicing factors for aberrant expression and AS events to associate them with NSCLC clinicopathological and prognostic data from patients using the online The Cancer Genome Atlas (TCGA) data. We then explored the role of the abnormally expressed m6a-related splicing factors in the regulation of the GAS events and constructed the risk signature of these factors to predict NSCLC prognosis after the gene ontology (GO) and the Kyoto Encyclopedia of Genes and Genomes (KEGG) pathway analysis. This study could provide a novel insight into the discovery of biomarkers in the prediction of NSCLC prognosis and possibly the underlying molecular mechanisms of NSCLC oncogenesis and development.

## Materials and Methods

### Data Download and Analysis

In this study, we first searched and downloaded differential gene expression profiles in LUAD and LUSC tissue specimens from The Cancer Genome Atlas (TCGA) database (https://portal.gdc.cancer.gov/). The corresponding clinicopathological data were subsequently downloaded from the University of California Santa Cruz database (https://xena.ucsc.edu/), which included 514 LUAD and 488 LUSC tissue samples. However, patients with incomplete clinical information and follow-up duration less than 30 days were excluded from our data analysis, resulting in 504 LUAD and 479 LUSC samples in this study. Moreover, the gene alternative splicing (GAS) events in LUAD and LUSC were download from TCGA Splice Seq (https://bioinformatics.mdanderson.org/TCGASpliceSeq/PSIdownload) and then calculated for the percent spliced in index (PSI) value, a quantifiable GAS indicator after the comparison of single and multiple samples between subgroups, that is, calculation of the percentage of GAS value for each GAS event, which was typically used to quantify GAS events according to a previous study (Lin and Krainer, 2019). We downloaded the contents that included seven main GAS types, that is, the exon skip (ES), retained intron (RI), alternate donor site (AD), alternate acceptor site (AA), alternate promoter (AP), alternate terminator (AT), and mutually exclusive exons (ME).

### Selection and Analysis of N6‐Methyladenosine RNA Methylation Regulatory Genes

In this study, we selected 13 m6A RNA methylation regulatory genes, that is, N6-adenosine-methyltransferase 70-kDa subunit (METTL3), methyltransferase-like 14 (METTL14), Wilms’ tumor-1 associated protein (WTAP), KIAA1429, RNA-binding protein 15 (RBM15), zinc finger CCCH domain-containing protein 13 (ZC3H13), YTH domain-containing protein 1 (YTHDC1), YTHDC2, YTH domain family, member 1 (YTHDF1), YTHDF2, heterogeneous nuclear ribonucleoproteins C1/C2 (HNRNPC), fat mass and obesity-associated protein (FTO), and m6A demethylase alkB homolog 5 (ALKBH5). We assessed their role in diagnosis, progression, and prognosis of LUAD and LUSC, that is, we first imported data on these m6A regulators into Cytoscape software [version 3.8.2 (Shannon et al., 2003)] and analyzed the data using the ClueGO plugin. After that, we performed the gene ontology (GO) and Kyoto Encyclopedia of Genes and Genomes (KEGG) pathway and network analysis, and the GO terms included the cellular component (CC), molecular function (MF), biological process (BP), an adjusted *p* < 0.05 as a statistically significant value. Afterward, we utilized the “LIMMA” package (Ritchie et al., 2015) to select and analyze the differentially expressed m6A RNA methylation regulatory genes in LUAD and LUSC tissue specimens using the cutoff value of |log2 fold change (FC)| ≥ 1 and adjusted *p*-value < 0.05. The “euclidean” and “ward.D2” methods were utilized to cluster the tumor samples, and the “p heat map” R package was used to plot the differential expression analysis results and cluster analysis results. Spearman’s correlation analysis was performed to analyze the correlation between the clusters and clinical traits. We also performed the univariate and multivariate Cox regression analyses to predict the association of these m6A RNA methylated regulatory genes with the overall survival of patients using the “survival” R package (Rizvi et al., 2019).

### Association of Gene Alternative Splicing Events With Overall Survival of Patients

We utilized the “WGCNA” (weighted gene co-expression network analysis) package (Langfelder and Horvath, 2008) to associate the GAS events with the overall survival of LUAD and LUSC patients. The WGCNA package is able to analyze thousands of the most varied genetic information to identify the sets of genes to associate with tumor phenotypes (Meng et al., 2019); thus, this tool allowed us to analyze the information regarding the GAS event data in association with clinical traits data and profile of m6A regulatory gene expressions, but avoided any unnecessary procedures for multiple hypothesis testing and corrections. In this analysis, we first estimated the standard scale-free network according to formula A to generate the adequate β value (appropriate soft threshold power). We then constructed the weighted adjacency matrix using the formula B and converted the data into a topological overlap matrix (TOM). After that, we utilized the dynamic tree cutting method according to the hierarchical clustering to identify the modules that highly correlated with GAS events. The GAS event then used 1-TOM as distance measurement with the depth (the cutoff value of 2) and the minimum size (the cutoff value of 60). After that, the highly similar modules were fused by clustering and height truncation of 0.3 according to previous studies (Niemira et al., 2019; Wan et al., 2018; Xie and Xie, 2019). Last, we performed the Spearman’s correlation and module eigengenes analysis of m6A regulator genes expression for association with the clinical traits and prognosis of 487 LUAD patients. Significant data on the association of this m6A regulator genes expression with clinicopathological data were further analyzed according to a previous study using the |correlation coefficients| between m6A regulators and GAS events module more than 0.4 and adjusted *p* < 0.05 (Langfelder and Horvath, 2008).

In the LUSC cohort of patient’s data, we selected and further analyzed the modules of the most significant correlation between clinical features and m6A regulator genes, that is, the |correlation coefficients| between m6A regulators and GAS events module more than 0.4 and adjusted *p* < 0.05. We then performed univariate and multivariate Cox Regression analyses to screen associate the GAS events with the overall survival of LUAD and LUSC patients.

The formula A: Aij = power (Sij, β) = |Smn|^β^ (i and j represent the GAS event of i and j, respectively, while m and n were the numbers of node connections, and β was the appropriate soft threshold power). The formula B: $TOMij=\frac{\sum_{u}AiuAju+Aij}{\min\left(Ki,Kj\right)+1-Aij}$ (i and j represent the GAS event of i and j, respectively, while the letter u represents clinical traits and prognostic information).

### The Gene Ontology Term and Kyoto Encyclopedia of Genes and Genomes Pathway Analyses of Overall Survival-Related Gene Alternative Splicing Genes

After screening the m6A-related GAS events, we performed the GO terms and KEGG pathways analysis of these overall survival-related GAS genes. Specifically, we imported data on the m6A-related GAS genes and m6A regulator genes into Cytoscape software (3.8.2) and analyzed them using the ClueGO plugin. After associating the overall survival of patients, we performed the GO terms and KEGG pathway analyses of these GAS events-related genes and the GO terms included the cellular component (CC), molecular function (MF), and biological process (BP) using an adjusted *p* < 0.05 as a statistically significant value according to a previous study (Amado et al., 2014). After that, we constructed the functional network of these corresponding genes using the Cytoscape software.

### Risk Model Construction

We constructed the risk model using the LASSO Cox regression analysis that could prevent any overfitting of the overall survival-related genes according to a previous study (Tang et al., 2017), and then performed the multivariate Cox regression analysis to predict the usefulness of these overall survival-related genes using the following formula:

J=1n∑i=1n(f(xi)−yi)2+λ‖w‖1 (the greater the value of J, the better the prediction value; the letter w indicates a globally optimal value of lost J).

$Risk\;score=\sum_{n=x}^{n}\text{coef}\left(\text{X}\right)*\text{PSI}\left(\text{X}\right)$ [Coef(X) is the coefficient of each GAS gene and PSI(X) is the PSI value of the AS genes].

According to the hazard ratio (HR) values after the multivariate Cox regression analysis, we classified the m6A-related prognostic AS events into protective/risky AS events (HR > 1 as a risk factor; HR < 1 as a protective factor), and showed the Sankey diagram that plotted is by “ggalluvial, dplyr, and ggplot2” R packages (Graedel, 2019; Soh et al., 2019). According to the median value of the risk score of the signature of each cohort, we divided the LUAD and LUSC cohorts into two subgroups, that is, the high- and low-risk groups. We then utilized the “survival” and “survminer” package to calculate the survival significance of the high-/low-risk group in these NSCLC patients. We performed the Kaplan–Meier survival analysis and receiver operating characteristic (ROC) curves to further verify the predictive ability of the risk signature using the “survivalROC” package and “Survival” package in R (Park et al., 2004). The concordance index (C-index) was used to validate and quantify the discrimination ability of the risk signature. At last, we performed the univariate and multivariate Cox regression analyses to assess whether these risk models and clinicopathological features were independent predictors for the survival of LUAD and LUSC patients.

### Predictive Nomogram Construction

After the univariate and multivariate Cox regression analyses, we used the “RMS” package to construct the nomogram of the independent risk factors (Zhang et al., 2019). We then performed the Wilcoxon rank-sum test to verify the association of the risk model with clinical characteristics (using an adjusted *p*-value < 0.05 as the statistical significance cutoff). For the construction of the predictive nomogram, we utilized the calibration curves to evaluate and validate the application ability of the nomogram performance.

### Statistical Analysis

The “Limma” R package was utilized to analyze the difference in gene expression profiles, while the “WGCNA” package was used to select m6A-related GAS events. Moreover, the univariate, LASSO and multivariate Cox regression analyses were performed to construct the risk signature, while the “Survival,” “survivalROC,” and “survminer” packages were utilized to verify the predictive efficacy of the risk signature in patients, while the area under the curve (AUC) value (ranged between 0.5 and 0.9) was used to assess the diagnostic ability of the risk signature (larger AUC value, better diagnostic value) (Verbakel et al, 2020). The Wilcoxon rank-sum and Spearman’s correlation tests were used to analyze the subgroup differences, while the “RMS” R package was utilized to plot the nomogram and calibrate the analytic data. The GO terms and KEGG pathways enrichment analysis were performed using Cytoscape software (3.8.2) and the “ggplot” R package was to plot the resulting data. All statistical analyses were performed using R software (version 3.6.1). A two-sided *p* value < 0.05 was considered statistically significant, while an adjusted *p*-value < 0.05 was applied as the threshold to avoid missing any significant changes.

## Results

### Characteristics of Lung Adenocarcinoma and Lung Squamous Cell Carcinoma Data and mRNA Splicing Events in The Cancer Genome Atlas Datasets

**FIGURE 1:**
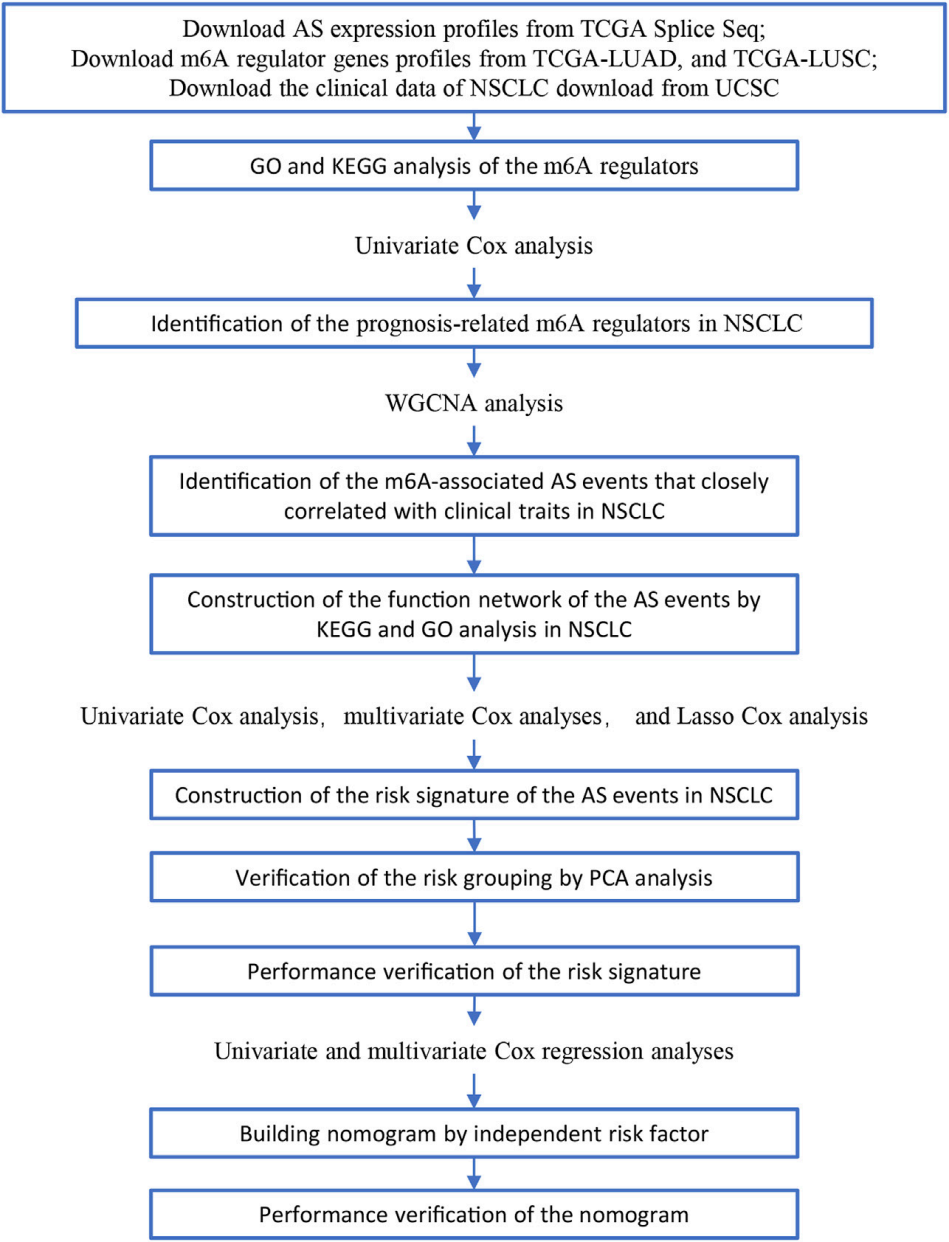
Illustration of the workflow in this study.

In this study, we searched and downloaded expression profiles of TCGA-LUAD and TCGA-LUSC from TCGA database (https://portal.gdc.cancer.gov/) and clinicopathological data from the University of California Santa Cruz database (https://xena.ucsc.edu/). We obtained 486 LUAD and 479 LUSC cases for our data analysis (Figure 1). In LUAD samples, there were 224 men and 262 women with a median age of 64.94 years old (arranged between 33 and 88 years). The patients were at the TNM stage of I/II (*n* = 381) and III/IV (*n* = 105) and 167 cases had lymph node tumor metastasis (Table 1). In LUSC cases, there were 353 men and 126 women with a median age of 64.294 years old (arranged between 39 and 90 years). The patients were at the TNM stage of I/II (*n* = 391) and III/IV (*n* = 88) and 169 cases had lymph node tumor metastasis (Table 1). Our data analyses identified a total of 43,948 mRNA splicing events in LUAD tissue samples and 46,020 mRNA splicing events in LUSC samples. The exon skip (ES) events were the most GAS events in both LUAD and LUSC groups of samples (Supplementary Figure S1).

**TABLE 1:** The univariate and multivariate cox regression analysis of clinicopathological data from TCGA-LUAD and LUSC.

Variables	Univariate analysis				Multivariate analysis			
	HR	HR.95L	HR.95H	*Adj-p*	HR	HR.95L	HR.95H	*Adj-p*
LUAD								
Age (≥65/<65)	1.111	0.823	1.500	4.92E-01	1.204	0.884	1.640	2.38E-01
Gender (female/male)	1.111	0.826	1.494	4.86E-01	1.077	0.791	1.467	6.37E-01
Smoking (yes/no)	0.864	0.440	1.697	6.72E-01	1.863	0.878	3.955	1.05E-01
TNM stage (I + II/III + IV)	1.635	1.422	1.881	***	1.564	1.187	2.062	***
T	1.548	1.296	1.848	***	1.066	0.865	1.315	5.48E-01
N	1.962	1.461	2.634	***	1.108	0.722	1.701	6.39E-01
M	2.181	1.302	3.653	**	0.740	0.355	1.543	4.21E-01
Risk score	1.441	1.346	1.543	***	1.390	1.281	1.508	***
LUSC								
Age ( ≥ 65/<65)	1.017	0.999	1.035	6.40E-02	1.028	1.007	1.050	*
Gender (female/male)	1.095	0.782	1.534	5.97E-01	1.225	0.834	1.800	3.01E-01
TNM stage (I + II/III + IV)	1.283	1.079	1.526	**	1.132	0.726	1.765	5.83E-01
T	1.352	1.121	1.630	**	1.434	1.083	1.897	**
N	1.170	0.952	1.438	1.35E-01	1.092	0.735	1.622	6.62E-01
M	2.455	0.905	6.659	7.77E-02	1.111	0.279	4.420	8.82E-01
Risk score	1.054	1.040	1.069	***	1.884	1.473	2.386	***

LUAD: lung adenocarcinoma; LUSC: lung squamous cell carcinoma; TNM: tumor-node metastasis; HR: hazard ratio.* represents *p* < 0.05; ** represents *p* < 0.05; *** represents *p* < 0.001.

### Association of N6-Methyladenosine RNA Methylation Regulatory Gene Expressions With Lung Adenocarcinoma and Lung Squamous Cell Carcinoma Prognosis

**FIGURE 2:**
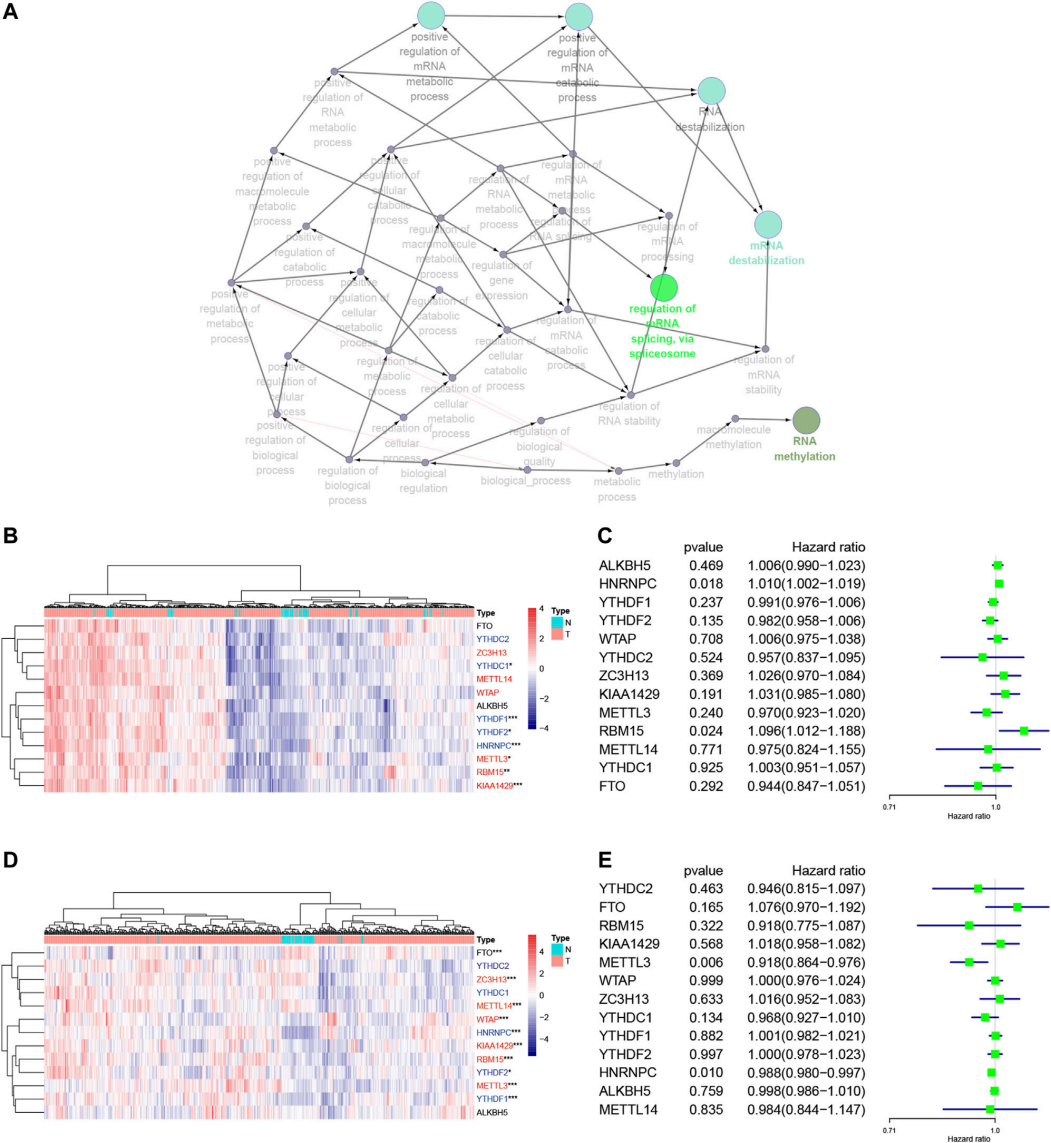
Association of m6A RNA methylation regulatory genes with NSCLC prognosis. **(A)** The GO terms and KEGG enrichment pathway analysis of the m6A regulator gene, the different colors represent the different pathways. **(B)** Differential expression of these 13 m6A RNA methylated regulator genes in LUAD (red: “writer”; blue: “readers”; black: “erasers”). **(C)** Forest plot of the univariate Cox regression analytic data. The 13 m6A RNA methylation regulators in LUAD were analyzed using the univariate Cox regression and the data are plotted using the forest plot. **(D)** Differential expression of these 13 m6A RNA methylated regulators in LUSC (red: “writer”; blue: “readers”; black: “erasers”). **(E)** Forest plot of the univariate Cox regression analysis. The 13 m6A RNA methylation regulators in LUAD were analyzed using the univariate Cox regression and the data are plotted using the forest plot. ***p < 0.001, **p < 0.01, and *p < 0.05. The KEGG, Kyoto Encyclopedia of Genes and Genomes; GO, Gene Ontology; LUAD, lung adenocarcinoma; LUSC, lung squamous cell carcinoma; m6A, N6-methyladenosine; N, Normal; T, Tumor.

We focused on 13 m6A RNA methylation regulatory genes, including METTL3, METTL14, WTAP, KIAA1429, RBM15, ZC3H13, YTHDC1, YTHDC2, YTHDF1, YTHDF2, HNRNPC, FTO, and ALKBH5. We performed the GO terms and KEGG pathway analyses and found that these m6A regulators were significantly enriched in the mRNA splicing spliceosome biology process, RNA methylation biology process, and RNA destabilization biology process (Figure 2A, Supplementary Table S1). We then performed the Wilcoxon signed-rank test and found that KIAA1429, HNRNPC, RBM15, METTL3, YTHDF1, YTHDF2, and YTHDC1 were differentially expressed between normal and LUAD tissues (Figure 2B). YTHDF1, YTHDF2, WTAP, KIAA1429, RBM15, METTL3, METTL14, FTO, HNRNPC, and ZC3H13 were differentially expressed between normal tissues and LUSC tissues (Figure 2D). Furthermore, KIAA1429, HNRNPC, RBM15, METTL3, YTHDF1, and YTHDF2 were all highly expressed in both LUAD and LUSC tissues (Figures 2B,D). The LUAD and LUSC patients were clustered into four groups according to the expression of m6A regulators in the heat map (Supplementary Figure S2A,B). The correlation analysis suggested that the differentially expressed m6A regulators were associated with status, smoking, TNM stage, and N stage in LUAD samples, and the differentially expressed m6A regulators were associated with status and age in LUSC samples (Table 2). These results suggested that m6A regulators play an important role in NSCLC development. The univariate Cox regression data revealed that HNRNPC and RBM15 expression were able to predict overall survival (OS) of LUAD patients (Figure 2C), while HNRNPC and METTL3 expression were associated with the OS of LUSC patients (Figure 2E); thus, these three m6A regulators genes were subjected to the subsequent analysis for association with OS of NSCLC patients as the splicing factors (Figures 2C,E).

**TABLE 2:** The correlation analysis of clinical traits and m6A clusters from TCGA-LUAD and LUSC.

		Overall	Cluster 1	Cluster 3	Cluster 4	Cluster 4	*p*
LUAD							
*n*		486	148	86	129	123	
Status (%)	Alive	366 (76.0)	123 (81.8)	61 (70.9)	76 (64.9)	106 (86.2)	***
	Dead	120 (24.0)	25 (18.2)	25 (29.1)	53 (35.1)	17 (13.8)	
Gender (%)	Female	262 (53.7)	74 (50.0)	52 (60.5)	69 (52.0)	67 (54.5)	0.483
	Male	224 (46.3)	74 (50.0)	34 (39.5)	60 (48.0)	56 (45.5)	
Age (%)	<65	215 (45.5)	49 (43.9)	40 (46.5)	65 (43.0)	61 (49.6)	0.71
	≥65	271 (54.5)	99 (56.1)	46 (53.5)	64 (57.0)	62 (50.4)	
Smoking (%)	No	13 (2.6)	1 (0.7)	7 (8.1)	1 (0.7)	4 (3.3)	**
	Yes	495 (97.4)	147 (99.3)	79 (91.9)	150 (99.3)	119 (96.7)	
Stage (%)	Stage I	263 (55.7)	85 (56.1)	38 (44.2)	60 (54.3)	80 (65.0)	*
	Stage II	118 (23.4)	38 (26.4)	25 (29.1)	33 (21.9)	22 (17.9)	
	Stage III	80 (15.7)	14 (9.5)	20 (23.3)	30 (19.9)	16 (13.0)	
	Stage IV	25 (5.1)	11 (8.1)	3 (3.5)	6 (4.0)	5 (4.1)	
T (%)	T1	165 (34.1)	59 (30.4)	26 (30.2)	30 (34.4)	50 (40.7)	0.104
	T2	256 (52.8)	74 (58.1)	47 (54.7)	73 (48.3)	62 (50.4)	
	T3	44 (9.1)	7 (5.4)	10 (11.6)	16 (10.6)	11 (8.9)	
	T4	21 (4.1)	8 (6.1)	3 (3.5)	10 (6.6)	0 (0.0)	
N (%)	N0	319 (67.3)	102 (69.6)	49 (57.0)	77 (65.6)	91 (74.0)	*
	N1	91 (17.7)	29 (18.9)	19 (22.1)	25 (16.6)	18 (14.6)	
	N2	70 (13.8)	12 (8.1)	18 (20.9)	26 (17.2)	14 (11.4)	
	N3	6 (1.2)	5 (3.4)	0 (0.0)	1 (0.7)	0 (0.0)	
M (%)	M0	460 (94.9)	136 (91.9)	83 (96.5)	123 (96.0)	118 (95.9)	0.274
	M1	26 (5.1)	12 (8.1)	3 (3.5)	6 (4.0)	5 (4.1)	
LUSC							
*n*		479	84	169	125	101	
Status (%)	Alive	293 (61.3)	60 (71.4)	108 (64.1)	63 (50.4)	62 (61.4)	*
	Dead	186 (38.8)	24 (28.6)	61 (35.9)	62 (49.6)	39 (38.6)	
Gender (%)	FEMALE	126 (26.5)	24 (28.6)	37 (22.4)	36 (28.8)	29 (28.7)	0.516
	MALE	353 (73.5)	60 (71.4)	132 (77.6)	89 (71.2)	72 (71.3)	
Age (%)	<65	166 (34.8)	37 (44.0)	61 (36.5)	30 (24.0)	38 (37.6)	*
	≥65	313 (65.2)	47 (56.0)	108 (63.5)	95 (76.0)	63 (62.4)	
Stage (%)	Stage I	235 (49.2)	35 (41.7)	83 (49.4)	60 (48.0)	57 (56.4)	0.176
	Stage II	156 (32.5)	33 (39.3)	54 (31.8)	46 (36.8)	23 (22.8)	
	Stage III	81 (16.9)	14 (16.7)	32 (18.8)	16 (12.8)	19 (18.8)	
	Stage IV	7 (1.5)	2 (2.4)	0 (0.0)	3 (2.4)	2 (2.0)	
T (%)	T1	109 (22.9)	16 (19.0)	34 (20.6)	32 (25.6)	27 (26.7)	0.567
	T2	281 (58.5)	51 (60.7)	103 (60.6)	67 (53.6)	60 (59.4)	
	T3	68 (14.2)	13 (15.5)	25 (14.7)	22 (17.6)	8 (7.9)	
	T4	21 (4.4)	4 (4.8)	7 (4.1)	4 (3.2)	6 (5.9)	
N (%)	N0	310 (64.8)	54 (64.3)	106 (62.9)	82 (65.6)	68 (67.3)	0.263
	N1	125 (26.0)	23 (27.4)	49 (28.8)	35 (28.0)	18 (17.8)	
	N2	39 (8.1)	6 (7.1)	14 (8.2)	6 (4.8)	13 (12.9)	
	N3	5 (1.0)	1 (1.2)	0 (0.0)	2 (1.6)	2 (2.0)	
M (%)	M0	472 (98.5)	82 (97.6)	169 (100.0)	122 (97.6)	99 (98.0)	0.264
	M1	7 (1.5)	2 (2.4)	0 (0.0)	3 (2.4)	2 (2.0)	

LUAD: lung adenocarcinoma; LUSC: lung squamous cell carcinoma; T: tumor; N: node; M: metastasis; * represents *p* < 0.05; ** represents *p* < 0.05; *** represents *p* < 0.001.

### Association of N6-Methyladenosine RNA Methylation Regulatory Genes With Lung Adenocarcinoma and Lung Squamous Cell Carcinoma Clinical Features

**FIGURE 3:**
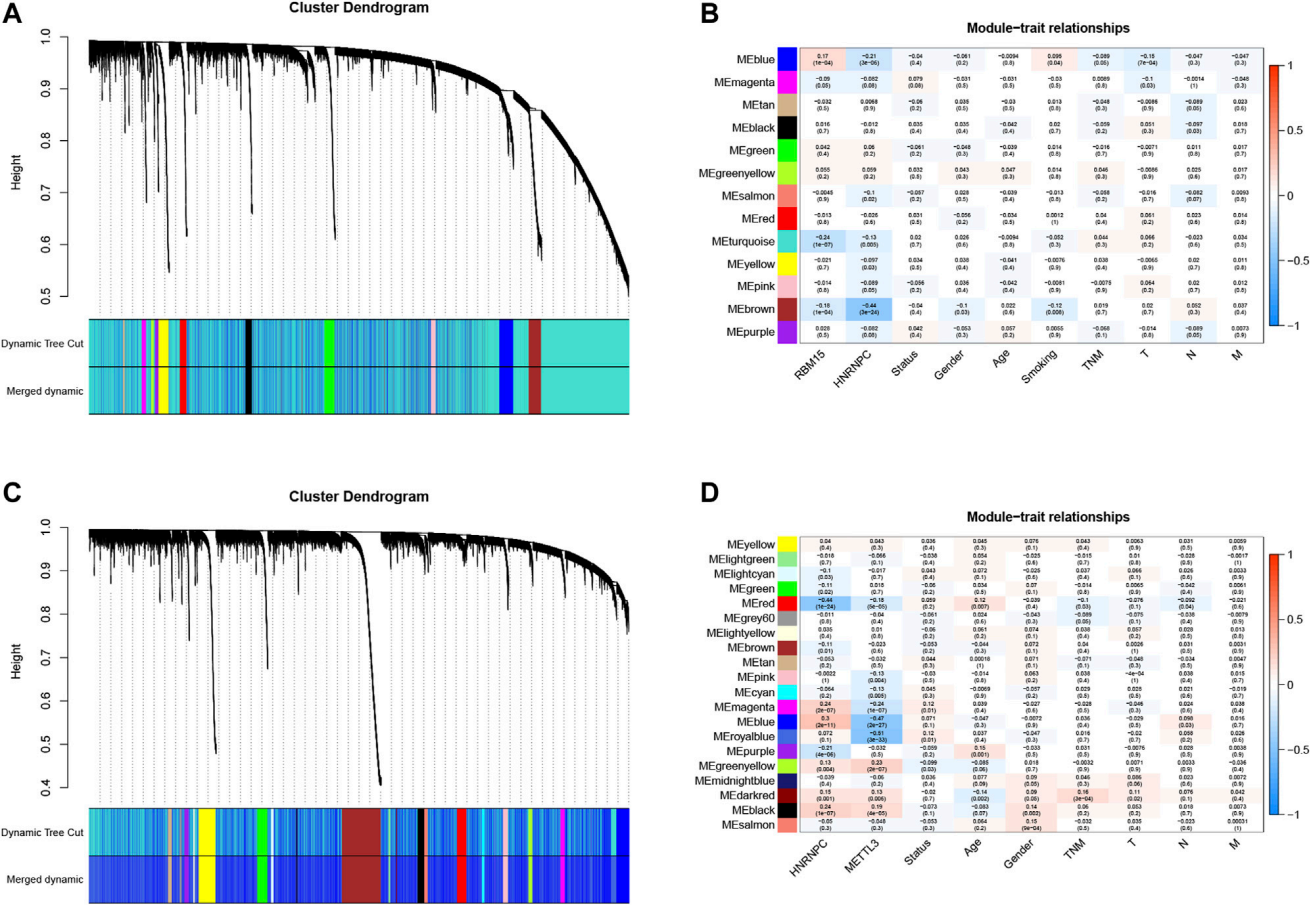
Identification of the AS events that are associated with clinical traits and the expression of m6A regulators. **(A)** Hierarchical cluster tree of the AS events in TCGA-LUAD samples. The tree indicates a unique TCGA-LUAD name and experiment identifier. **(B)** Association of the AS events with gender, age, LUAD lymphatic infiltration, tumor status, and pathological stages. The number represents the Pearson correlation values between the module and the features. The number in parenthesis is the *p*-value, while the numbers in the color column represent the intensity of the correlation in the table (red = 1, blue = − 1). **(C)** Hierarchical clustering tree of the AS events in TCGA-LUSC samples. The tree indicates a unique name and experiment identifier marked TCGA-LUSC. **(D)** Association of the AS events with gender, age, LUSC lymphatic vessel invasion, tumor status, and pathological stages. The number represents the Pearson correlation values between the module and the features The number in parenthesis is the *p*-value, while the numbers in the color column represent the intensity of the correlation in the table (red = 1, blue = 1). T, tumor scope; N, degree of diffusion to lymph nodes; M, there is a transfer; m6A, N6-methyladenosine, LUAD, lung adenocarcinoma; LUSC, lung squamous cell carcinoma; AS, alternative splicing.

After that, we correlated the GAS events with the weighted gene co-expression network, and they were consistent with the scale-free network (Supplementary Figure S3). The hierarchical clustering analysis of the samples using the Euclidean distance showed log10-transformed RNA-seq fractional counts (Supplementary Figure S4), while the dynamic tree cutting method identified the modules with a similar expression spectrum and combine similar modules (Figures 3A,C, Supplementary Figure S5). We then utilized the “WGCNA” package to analyze the GAS events and Spearman’s correlation test to associate the expression of m6A regulator genes with clinical traits. The data showed that the MEbrown module was significantly associated with expression of the m6A regulator genes (RBM15, *p* = 3e-24, the |correlation coefficient| = −0.44), gender (*p* = 0.03, the coefficient correlation = −0.1), and tobacco smoking (*p* = 0.006, the coefficient correlation = −0.12) of LUAD patients (Figure 3B, Supplementary Figure S6A). Furthermore, the MEred, MEblue, and MEroyalblue modules were significantly associated with expression of the m6A regulator genes (MEred, HNRNPC with an adj *p* = 1e-24 and the coefficient correlation = −0.44; MEblue, HNRNPC with an adj *p* = 2e-27 and the coefficient correlation = −0.47; MEroyalblue, HNRNPC with an adj *p* = 3e-33 and the coefficient correlation = −0.51). These three modules were also associated with the age of patients (MEred, adj *p* = 0.007 and the coefficient correlation = 0.12), the TNM stage (MEred, adj *p* = 0.03 and the coefficient correlation = −0.1), and the N stage (MEblue, adj *p* = 0.04 and the coefficient correlation = −0.092; MEroyalblue, adj *p* = 0.03 and the coefficient correlation = 0.098; in Figure 3D, Supplementary Figure S6B). These results suggested that the m6A-related AS events in the MEred, MEblue, and MEroyalblue modules could predict NSCLC development and lymph node metastasis, while the age of patients might also affect the m6A-related AS events.

**FIGURE 4:**
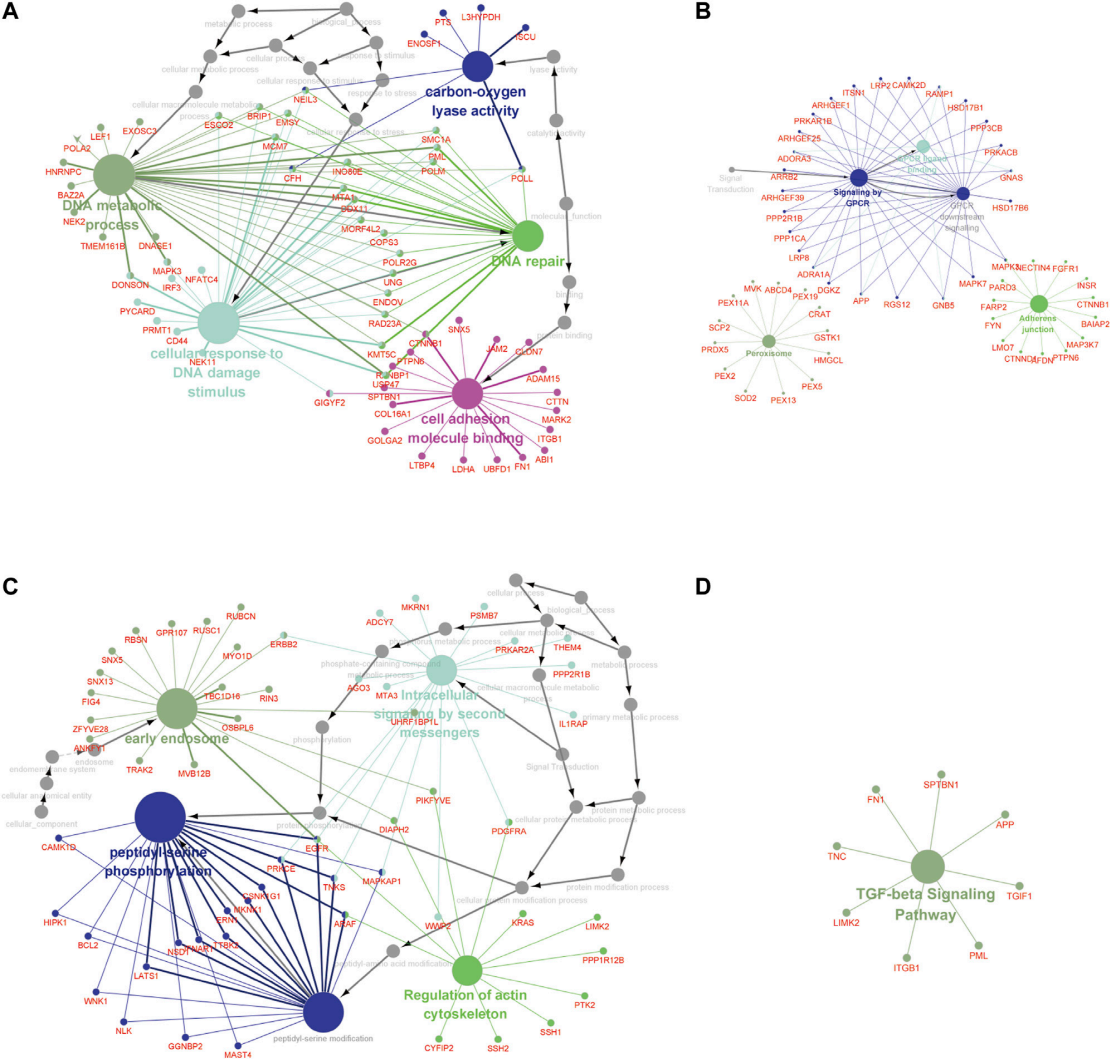
The GO terms and KEGG pathway analyses of genes from m6A-related AS events in NCSLC. The different colors represent the different pathways **(A, B)** in the regulation network. The GO terms **(A)** and KEGG pathways enrichment **(B)** analysis of the m6A-related prognostic AS genes in LUAD. **(C, D)** The regulation network. The GO terms **(C)** and KEGG pathways enrichment **(D)** analysis of the m6A-related prognostic AS genes in LUSC. The KEGG, Kyoto Encyclopedia of Genes and Genomes; GO, Gene Ontology; LUAD, lung adenocarcinoma; LUSC, lung squamous cell carcinoma; AS, alternative splicing.

Furthermore, the MEbrown module included 1.102 GAS events, and the MEred, MEblue, and MEroyalblue modules included 1.5150 GAS events. The most significant enrichment of the GO terms and KEGG pathway analysis of LUAD cohort revealed the m6A-related AS events were significantly enriched in the GPCR signaling pathway, DNA metabolic process, DNA repair, cellular response to DNA damage stimulus, carbon–oxygen lyase activity, and cell adhesion molecule binding pathways, while the m6A-related AS events in LUSC cohort were significantly enriched in the TGF-beta signaling pathway, peptidyl-serine phosphorylation, peptidyl-serine modification, regulation of actin cytoskeleton, intracellular signaling by second messengers, and early endosome pathway (adj *p* < 0.001; Figure 4, Supplementary Table S2).

### Association of N6-Methyladenosine-Related Alternative Splicing Events With Lung Adenocarcinoma and Lung Squamous Cell Carcinoma Prognosis

**FIGURE 5:**
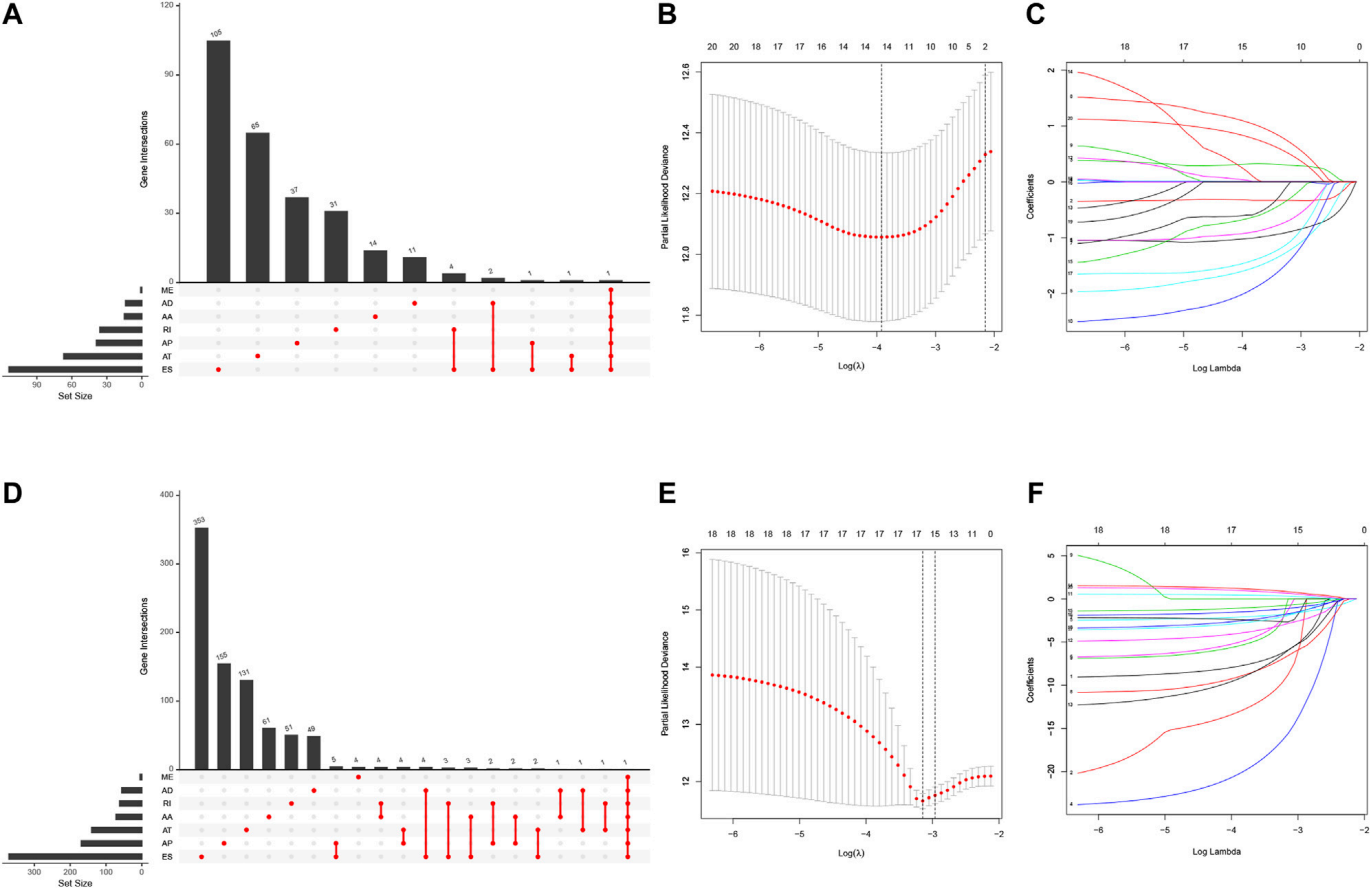
Identification of NSCLC prognosis-related AS events. **(A)** The prognostic upset plot. The data exhibit the m6A-related prognostic AS events in LUAD. **(B, C)** The LASSO Cox analysis. These 13 m6A-related AS events associated with LUAD prognostics and the optimal values of the penalty parameter were assessed using the 10-round cross-validation. **(D)** The prognostic upset plot. The data exhibit the m6A-related prognostic AS events in LUSC. **(E, F)** The LASSO Cox analysis. These 15 m6A-related AS events associated with LUSC prognostics and the optimal values of the penalty parameter were determined by the 10-round cross-validation. LUAD, lung adenocarcinoma; LUSC, lung squamous cell carcinoma; m6A, N6-methyladenosine; OS, overall survival: AS, alternative splicing.

We first performed the univariate Cox regression analysis to identify m6A-related AS events for association with LUAD and LUSC prognosis. We found 292 prognostic AS events in LUAD and 922 prognostics AS events in LUSC (*p* < 0.05; Figures 5A,D, Supplementary Table S3). The LASSO Cox regression analysis confirmed 13 of the prognostic AS events in LUAD (Figures 5B,C) and 15 in LUSC (Figures 5E,F), while the multivariate Cox regression analysis further confirmed seven of the prognostic AS events in LUAD and 14 in LUSC (Supplementary Table S4).

### Non–Small Cell Lung Cancer Prognosis-Related Gene Alternative Splicing Events Signature

**FIGURE 6:**
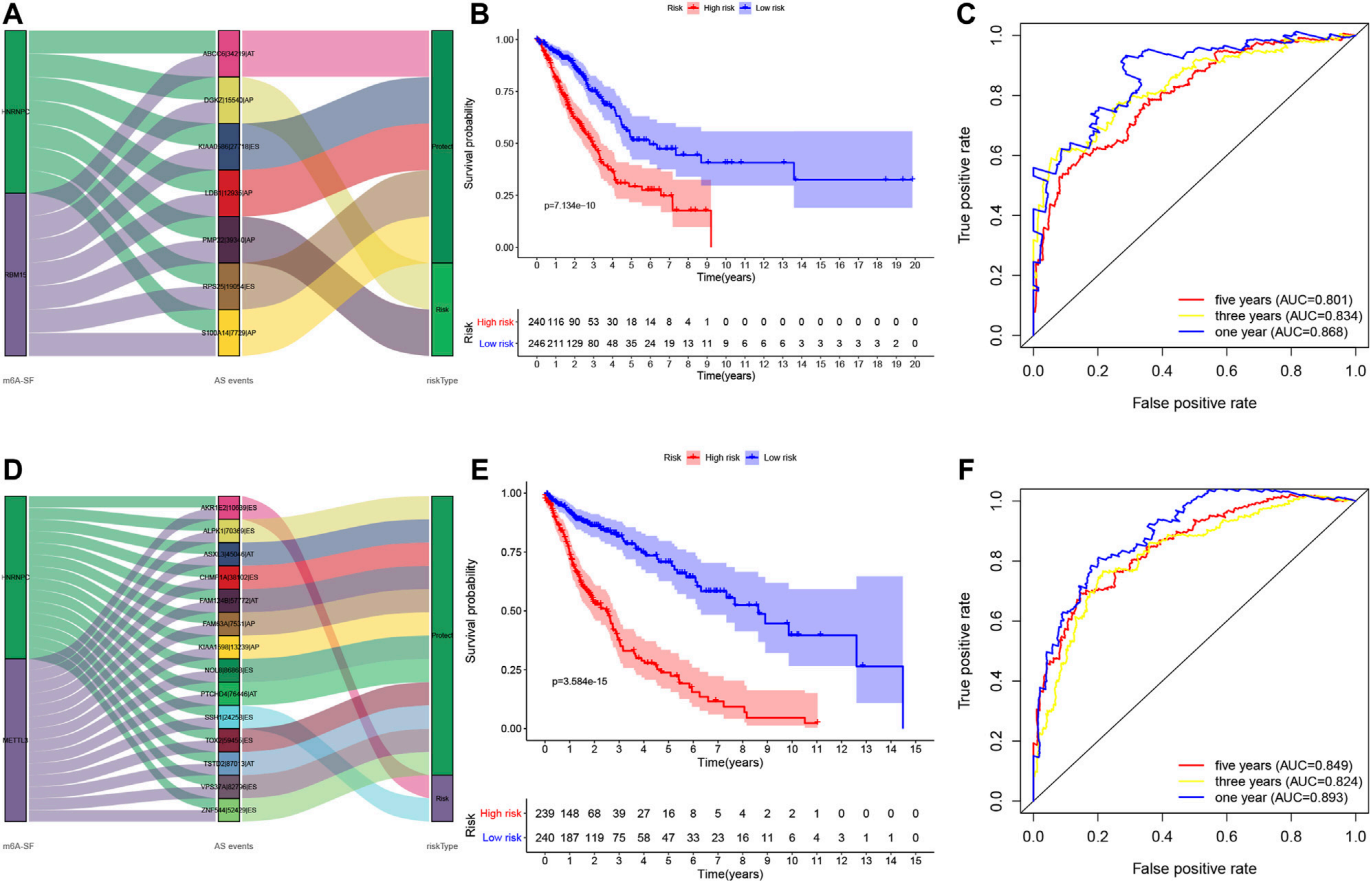
Construction of the prognosis-related AS events signature. **(A)** The Sankey diagram. The data show the potential prognostic value of the m6A-related prognostic AS events in LUAD. **(B)** Kaplan–Meier curves stratified by high- and low-risk groups of LUAD. **(C)** The ROC curves in LUAD. **(D)** The Sankey diagram. The data show the potential prognostic value of the m6A-related prognostic AS events in LUSC. **(E)** Kaplan–Meier curves stratified by high- and low-risk groups of LUSC. **(F)** The ROC curves in LUSC. LUAD, lung adenocarcinoma; LUSC, lung squamous cell carcinoma; ROC, receiver operating characteristic curve.

We utilized these seven and 14 AS genes in LUAD and LUSC, respectively, to further construct the LUAD and LUSC risk signature (Supplementary Table S5). The Sankey diagram shows that DGKZ|15540|AP and PMP22|39340|AP were the risky m6A-related AS events in LUAD (HR > 1), whereas ABCC6|34219|AT, KIAA0586|27718|ES, LDB1|12935|AP, RPS25|19054|ES, and S100A14|7729|AP were the protective m6A-related AS events in LUAD (HR < 1) (Figure 6A, Supplementary Table S5). Furthermore, AKR1E2|10639|ES and SSH1|24258|ES were the risky m6A-related AS events in LUSC (HR > 1), whereas ALPK1|70369|ES, FAM63A|7531|AP, CHMP1A|38102|ES, TSTD2|87013|AT, KIAA1598|13239|AP, ASXL3|45046|AT, VPS37A|82796|ES, TOX2|59455|ES, ZNF544|52429|ES, NOL8|86863|ES, FAM124B|57772|AT, and PTCHD4|76446|AT were the m6A-related protective AS events in LUSC (HR < 1; Figure 6D, Supplementary Table S5). We then divided LUAD and LUSC patients into high- and low-risk groups according to their risk scores (high-risk LUAD group, *n* = 240; high-risk LUSC group, *n* = 239; low-risk LUAD group, *n* = 246; and low-risk LUSC group, *n* = 240; Supplementary Table S6). The Kaplan–Meier curve analyses showed that the high-risk group had a poorer OS than the low-risk group (*p* < 0.001; Figures 6B,E). The ROC analysis revealed that the AUC values were 0.868, 0.834, and 0.801 for 1-, 3-, and 5-year OS of LUAD, respectively, while the AUC values were 0.893, 0.824, and 0.849 for 1-, 3-, and 5-year LUSC OS, respectively (Figures 6C,F). The concordance index (C-index) of OS was 0.847 [95% confidence interval (CI): 0.788–0.847] in LUAD and 0.832 in LUSC (95% CI: 0.788–0.877; Supplementary Table S7).

**FIGURE 7:**
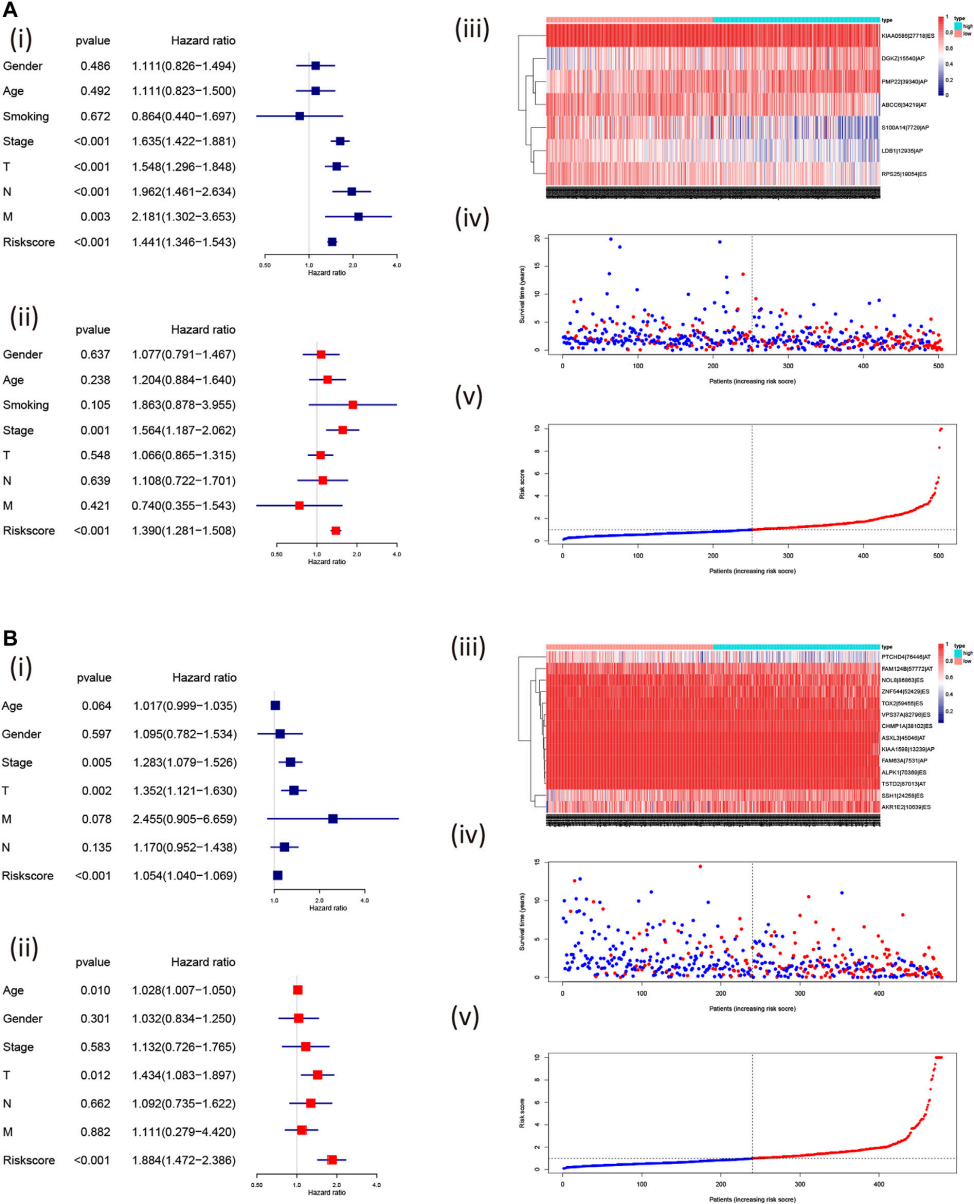
Assessment of the independent prognostic value using the risk scores. The univariate and multivariate Cox regression analyses of the risk signature in LUAD **(A)**; i, univariate and ii, multivariate Cox regression analyses and in LUSC **(B)**; i, univariate and ii, multivariate Cox regression analyses risk signature. The visualized gene percent spliced index value in LUAD (**A**; iii) and LUSC (**B**; iii) and risk scores were associated with NSCLC survival [LUAD: **(A)**: iv, v; LUSC: **(A)**: iv, v; the red dot or line represents the deceased, while the blue dot or line represents alive]. LUAD, lung adenocarcinoma; LUSC, lung squamous cell carcinoma; PSI, percent spliced in index.

Furthermore, we found that the risk signature (univariate Cox analysis, HR: 1.543, 95% CI: 1.441–1.346; *p* < 0.001; multivariate Cox analysis, HR: 1.390, 95% CI: 1.281–1.508; *p* < 0.001) and the TNM stage (univariate Cox analysis, HR: 1.635, and 95% CI: 1.422–1.881; *p* < 0.001; multivariate Cox analysis, HR: 1.564, 95% CI: 1.187–2.062; *p* = 0.001) were independent prognostic factors of LUAD, while the risk signature (univariate Cox analysis, HR: 1.054, 95% CI: 1.040–1.069; *p* < 0.001; multivariate Cox analysis, HR: 1.884, 95% CI: 1.472–2.386; *p* < 0.001) and T stage (univariate Cox analysis, HR: 1.352, 95% CI: 1.121–1.630; *p* = 0.002; multivariate Cox analysis, HR: 1.434, 95% CI: 1.083–1.897; *p* = 0.012) were independent prognostic factors in LUSC (Figures 7A,B, Table 2). The risk curve and scatterplot of the risk score and survival of each NSCLC sample and the heat map of these AS genes in NSCLC samples are shown in Figures 7Aiii–v, iii–v.

### Association of These Prognostic Signatures With Non–Small Cell Lung Cancer Clinicopathologies

**FIGURE 8:**
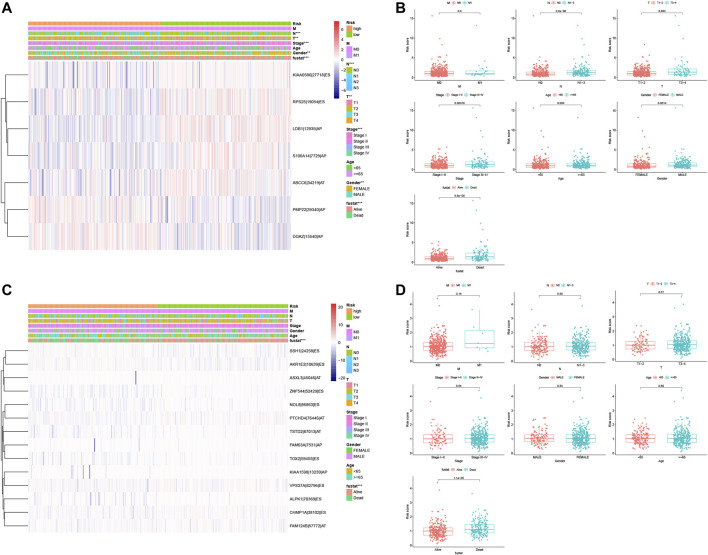
Association of the risk signature with clinicopathological features from both LUAD and LUSC. **(A)** Heat map of different clinicopathological characteristics from high- *vs.* low-risk LUADs stratified by percent spliced index (PSI) of the AS events. **(B)** Association of the risk scores with clinicopathological features from LUAD. **(C)** Heat map of different clinicopathological features of from high- *vs.* low-risk stratified by the PSI of the AS events. **(D)** Association of the risk scores with clinicopathological features from LUSC. ***p < 0.001, **p < 0.01, and *p < 0.05. AS, alternative splicing; LUAD, lung adenocarcinoma; LUSC, lung squamous cell carcinoma; PSI, percent spliced in index.

After that, we associated these prognostic signatures with NSCLC clinicopathologies and found that the LUAD risk signature was associated with the gender of patients and tumor T, N, and TNM stages (adj *p* < 0.05; Figures 8A,C, Table 3), although there was no association occurred between the LUSC risk signature and clinical features (Table 3). Furthermore, male (*n* = 224), TNM stage III–IV (*n* = 105), N stage 1–3 (*n* = 167), and T stage 3–4 (*n* = 65) of LUAD patients in had significantly higher risk scores than female (*n* = 262), TNM stage I–II (*n* = 381), N stage 0 (*n* = 319), and T stage 1–2 (*n* = 421; all adj *p* < 0.05; Figure 8, Table 3). Older age (*n* = 271) and M1 stage (*n* = 26) of LUAD patients also had the higher risk scores (all adj *p* > 0.05; Figure 8B, Table 3). Notably, male (*n* = 353), TNM stage III–IV (*n* = 88), N stage 1–3 (*n* = 169), M1 stage (*n* = 7), older age (*n* = 313), and T stage 3–4 (*n* = 89) of LUSC patients also had higher risk scores than those of the corresponding subgroups, but the differences did not appear statistically significant (Figure 8D).

**TABLE 3:** Clinicopathological features from LUAD and LUSC subgroups stratified by the AS events signature.

	LUAD	*Adj-p*	LUSC	*Adj-p*
Gender		*		8.96E-01
Male	224		353	
Female	262		126	
Age		1.38E-01		9.50E-01
<65	215		166	
≥65	271		313	
TNM stage		***		5.04E-01
I	263		235	
II	118		156	
III	80		81	
IV	25		7	
Tumor (T)		**		7.25E-01
T1	165		110	
T2	256		280	
T3	44		68	
T4	21		21	
Lymph node (N)		***		6.30E-01
N0	319		310	
N1	91		125	
N2	70		39	
N3	6		5	
Metastasis (M)		5.17E-01		9.96E-01
M0	460		472	
M1	26		7	
Status		***		**
Dead	120		293	
Alive	366		186	
Total case	486		479	

LUAD: lung adenocarcinoma; LUSC: lung squamous cell carcinoma; TNM: tumor-node metastasis; *** represents *p* < 0.001; ** represents *p* < 0.01; * represents *p* < 0.05.

### Usefulness of the Predictive Nomogram in Non–Small Cell Lung Cancer

**FIGURE 9:**
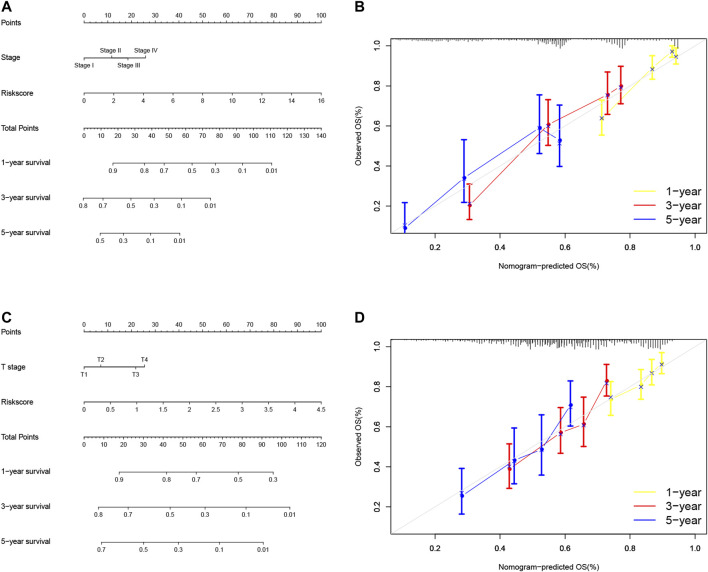
Nomogram prediction of overall survival (OS) of LUAD and LUSC patients. **(A)** Construction of AS clinicopathological nomograms using AS risk signature and pathological stage. The nomogram was then used to predict the 1-, 3-, and 5-year OS of LUAD patients. **(B)** The nomogram AS clinicopathological nomogram calibration plot in LUAD. It predicts 1-, 3-, and 5-year prognoses. **(C)** Construction of AS clinicopathological nomograms using AS risk signature and T stage. The nomogram was then used to predict 1-, 3-, and 5- OS of LUSC patients. **(D)** The AS clinicopathological nomogram calibration plot. It predicts the 1-, 3-, and 5-year prognosis of LUSC. AS, alternative splicing; LUAD, lung adenocarcinoma; LUSC, lung squamous cell carcinoma; OS, overall survival.

So far, we showed the risk signature and TNM stage as independent prognostic predictors in LUAD and the risk signature and T stage as independent prognostic predictors in LUSC. We thus, constructed the nomogram using these parameters to assess and apply this risk model for NSCLC (Figures 9A,C) and verified the calibration curves of the nomogram (Figures 9B,D). We were able to use the numerous values of this risk model to predict the 1-, 3-, and 5-year survival of NSCLC patients.

## Discussion

In the current study, we analyzed the aberrant expression of 13 m6A regulatory genes and related GAS events to construct a risk gene signature to predict the overall survival of NSCLC patients. We found that a number of them were highly expressed in LUAD or LUSC tissues vs. their normal ones, which could be used to predict the survival of patients. Furthermore, we found 43,948 mRNA splicing events in LUAD and 46,020 in LUSC and m6A regulators could regulate mRNA splicing. We then constructed the NSCLC prognosis-related AS events signature and divided the patients into high- *vs.* low-risk groups using seven and 14 AS genes in LUAD and LUSC, respectively. The data showed that DGKZ|15540|AP and PMP22|39340|AP were the risky m6A-related AS events in LUAD, whereas ABCC6|34219|AT, KIAA0586|27718|ES, LDB1|12935|AP, RPS25|19054|ES, and S100A14|7729|AP were the protective m6A-related AS events in LUAD. Similarly, AKR1E2|10639|ES and SSH1|24258|ES were the risky m6A-related AS events in LUSC, whereas ALPK1|70369|ES, FAM63A|7531|AP, CHMP1A|38102|ES, TSTD2|87013|AT, KIAA1598|13239|AP, ASXL3|45046|AT, VPS37A|82796|ES, TOX2|59455|ES, ZNF544|52429|ES, NOL8|86863|ES, FAM124B|57772|AT, and PTCHD4|76446|AT were the m6A-related protective AS events in LUSC. Further analyses showed that the LUAD risk signature was associated with the gender of patients and tumor T, N, and TNM stages. In addition, the risk signature and TNM stage were independent prognostic predictors in LUAD and the risk signature and T stage were independent prognostic predictors in LUSC. In conclusion, our current study demonstrated the usefulness of this AS prognostic signature in the prediction of LUAD and LUSC prognosis. Further study will verify this AS signature in a prospective dataset from NSCLC patients.

M6A modification and GAS occur most commonly in mRNA transcripts and their alterations play an important role in the development and progression of human cancers (Cherry and Lynch, 2020; Sun et al., 2019). Accumulated evidence suggests that m6A regulators-mediated gene methylation played a critical role in NSCLC development (ref); however, the underlying molecular mechanisms of m6A regulator actions in cancer development remain to be fully elucidated. Recently, the m6A regulators have been shown to act as an important splicing factor during GAS events (Kasowitz et al., 2018; Yoshimi et al., 2019; Geng et al., 2020), although research of the m6A regulator regulating AS events is still in the early stage in the field of cancer research, including lung cancer. Therefore, our current study conducted the GO terms and KEGG pathway analyses of these m6A and related GAS events in NSCLC and found that m6A regulators were significantly enriched in the regulation mRNA splicing spliceosome biology process. We also found that the expression of some of them, including METTL3, HNRNPC, and RBM15, could predict NSCLC prognosis, although the hazard ratios suggest that their prediction of NSCLC survival might be marginal. A previous study from Sun et al. (2020) showed the usefulness of the m6A regulators as the risk signature in LUAD (AUC, 0.65–0.82). Our results also suggested that m6A regulators play an important role in NSCLC development. Other previous studies (Lee et al., 2010; Gruber et al., 2016; Li et al., 2020) reported that HNRNPC was an RNA-binding protein (the “reader”), which could regulate RNA splicing, 3-terminal processing, and translation (Gruber et al., 2016; Lee et al., 2010; Li et al., 2020). HNRNPC overexpression was observed in a variety of human cancers, including lung cancer (Park et al., 2012) HNRNPC, as a protein-coding gene, could also interact with KHSRP to activate the IFN-α-JAK-p-STAT1 signaling pathway and promoted NSCLC cell proliferation, migration, and invasion (Yan et al., 2019). It can also regulate the GAS as an “m6A switcher” (Alarcón et al., 2015; Dai et al., 2018; Li et al., 2017; Wang et al., 2020). Furthermore, RBM15, as a “writer,” can bind to METTL3 and WTAP and direct them to specific RNA sites for m6A modification (Wang et al., 2020), although it does not possess any catalytic functions (Chen et al., 2019). RBM15 was also shown to interact with the METTL3 complex and depletion of these adapters could also reduce the m6A level (Pendleton et al., 2017). Further investigation of RBM15 and GAS events revealed that RBM15 was able to bind to specific intron regions to recruit the splicing factor SF3B1AS (Zhang et al., 2015). In addition, *METTL3*, containing highly conserved sequences, is the most important component of the m6A methyltransferase complex and was shown to be an S-adenosyl methionine (SAM)–binding protein and catalyze m6A modification (Wang et al., 2016). METL3 expression was high in LUAD and promoted the translation of the epidermal growth factor receptor (EGFR) mRNA and hippo pathway effector TAZ mRNA in lung cancer cells, for induction of cell growth, survival, and invasion (Lin et al., 2016). METTL3 was also shown to interact with GAS events of the skipped exons and alternative first exon (Alarcón et al., 2015), and METTL3 dysregulation was reported to indeed affect GAS events (Katz et al., 2015; Liu et al., 2014). METTL3 silence significantly affected gene expression and alternative splicing patterns, leading to modulation of the p53 pathway and cell apoptosis (Dominissini et al., 2012). Taken altogether, these three m6A RNA methylation regulatory genes were important in the regulation of GAS events in NSCLC.

Indeed, AS events is an important mRNA modification process and produce a large number of mRNA and protein isoforms with different regulatory functions (Bonnal et al., 2020; Liu et al., 2020). The prognostic value of the AS events in NSCLC has well been documented, for example, Zhao et al. (2020) built a predictive model of aberrant AS events and predicated NSCLC prognosis. Indeed, alternation in splicing factor expression could alter many AS events in NSCLC (Coomer et al., 2019). For instance, QKI was shown to one of the most downregulated splicing factors in NSCLC, while QKI-5 was able to competitively bind to NUMB with SF1 protein to induce the NUMB exon 11 skip and, therefore, inhibited the Notch signaling (Zong et al., 2014; de Miguel et al., 2016). In lung cancer, QKI expression was significantly reduced, increasing in the abnormal splicing of num exon 11 to, in turn, activate the Notch signaling pathway and tumor cell proliferation (Zong et al., 2014; de Miguel et al., 2016). The AS events also influenced p53 expression in NSCLC and MDM2-B, an AS product of MDM2, was able to promote p53-independent cell growth and inhibition of apoptosis (Coomer et al., 2019). In this regard, the AS events are important in NSCLC development and progression (Bonnal et al., 2020; Sciarrillo et al., 2020).

Furthermore, the weighted gene co-expression network analysis (WGCNA) is a widely used data mining method, especially used for studying the biological networks based on pairwise correlations between variables (Langfelder and Horvath, 2008). In the current study, we used WGCNA to select the AS events that are highly correlated with the NSCLC survival-related m6A RNA regulators. After that, we performed the GO and KEGG pathways enrichment analysis to identify genes of m6A-related AS events to significantly participate in gene pathways that play an important role in NSCLC tumorigenesis, progression, drug sensitivity, and metastasis. Indeed, some of the abnormal AS events were associated with drug sensitivity and resistance of NSCLC (Motegi et al., 2019; Pilié et al., 2019) as well as cell adhesion molecule binding process (Song et al., 2013; Hintermann and Christen, 2019). Our KEGG analysis showed that the genes in the m6A-related AS events significantly participated in the GPCR signaling in LUAD, and the latter is mediated by three major G protein subclasses and each subclass also has multiple proteins that are products due to the AS events (Kang et al., 2015; Kallifatidis et al., 2020). Similarly, we found the m6A-related AS events in the TGF-β signaling in LUSC. The TGF-β signaling pathway was frequently downregulated in human cancers (Syed, 2016), whereas this pathway activation could also promote tumorigenesis, metastasis, and chemoresistance (Colak and Ten Dijke, 2017; Zi, 2019). In this regard, genes of the m6A-related AS event-led activation of the TGF-β signaling could promote LUSC tumorigenesis. However, further study is needed to confirm this speculation.

In addition, in our current study, we constructed the risk signature using these altered genes in m6A and AS events to associate with NSCLC prognosis, and the AUC of the ROC curves showed the sensitivity and specificity of LUAD and LUSC, respectively, which are better than other recent studies (Zhao et al., 2020) (Liu et al., 2020). In these risk signatures, a previous study showed that S100A14 overexpression was able to promote LUAD cell migration and invasion (Ding et al., 2018). In all recent studies of the AS events in NSCLC, Li et al. (2017) were the first to construct an AS risk signature for the prediction of NSCLC prognosis, while Zhao et al. (2020) constructed the AS risk signature stratified by gender of patients. Liu et al. (2020) formed an AS signature for LUSC. Our current study also explored abnormal expression of the splicing factors in NSCLC as well as the C-index (Supplementary Table S7). However, our current study does have some limitations, for example, the AS events database is relatively simple and lacks all other relevant datasets for us to verify our data. In addition, the relationship of m6A regulators with the AS events and the mechanism by which they play a role in NSCLC development remain; thus, more studies are needed to clarify the true biological role of the AS events in NSCLC tumorigenesis.

## Conclusions

Our current study assessed the role of m6A-related AS events in NSCLC as a signature in the prediction of NSCLC prognosis. The current study revealed the regulation of AS events by some key m6A regulators may play an important role in NSCLC development and progression. This study might provide a novel insight into the mechanism of NSCLC tumorigenesis, which may lead to novel strategies in future control of NSCLC.

## Data Availability

The data that support the findings of this study are openly available in the TCGA-LUSC and TCGA-LUAD database (https://portal.gdc.cancer.gov/).

## Supplementary Material

SUPPLEMENTARY FIGURE S1 | The upstate diagrams of LUAD and LUSC. **(A)** The upstate diagram of alternative splicing events in LUAD. **(B)** The upstate diagram of alternative splicing events in LUSC. LUAD, lung adenocarcinoma; LUSC, lung squamous cell carcinoma.

SUPPLEMENTARY FIGURE S2 | **(A)** The cluster dendrogram of the LUAD patients. **(B)** The cluster dendrogram of the LUSC patients. LUAD, lung adenocarcinoma; LUSC, lung squamous cell carcinoma.

SUPPLEMENTARY FIGURE S3 | Cluster analysis of samples in LUAD **(A)** and LUSC **(B)** in the detection of the outliers. The white-to-red linear gradient color indicates the association with the corresponding clinical variables, while the gray indicates missing data. LUAD, lung adenocarcinoma; LUSC, lung squamous cell carcinoma.

SUPPLEMENTARY FIGURE S4 | Bioinformatical analysis of AS modules Associated with m6A RNA methylation regulatory genes. **(A)** The eigengene dendrogram and heat map. They identify groups of the correlated eigengenes termed meta-modules in LUAD. **(B)** Module–trait association in LUAD. Each row corresponds to a module eigengene, column to a trait. Each cell contains the corresponding correlation and *p*-value. The table is color-coded by correlation according to the legend. **(C)** The eigengene dendrogram and heat map. They identify groups of the correlated eigengenes termed meta-modules in LUSC. **(D)** Module–trait associations in LUSC. Each row corresponds to a module eigengene, column to a trait. Each cell contains the corresponding correlation and *p*-value. The table is color-coded by correlation according to the legend. LUAD, lung adenocarcinoma; LUSC, lung squamous cell carcinoma.

SUPPLEMENTARY FIGURE S5 | The parameter of the adjacency function in the weighted gene correlation network analysis algorithm. **(A)** Analysis of the soft threshold power and the average connectivity of various soft threshold powers in LUAD. **(B)** Analysis of the soft threshold power and the average connectivity of the various soft threshold powers in LUSC. LUAD, lung adenocarcinoma; LUSC, lung squamous cell carcinoma.

SUPPLEMENTARY FIGURE S6 | Scatterplot of the gene or clinical traits significance (the y-axis) vs. module membership (the x-axis) in the most significant module of LUAD **(A)** and LUSC **(B)**. In modules related to a trait of interest, genes with high module membership often also have had high gene significance. LUAD, lung adenocarcinoma; LUSC, lung squamous cell carcinoma. ADDIN
