# Supplementary figures and images for: N6-Methyladenosine RNA Methylation Regulator-Related Alternative Splicing (AS) Gene Signature Predicts Non–Small Cell Lung Cancer Prognosis
Source: Front Mol Biosci. 2021 Jun 11;8:657087. doi: 10.3389/fmolb.2021.657087 (PMC8226009; doi:10.3389/fmolb.2021.657087)

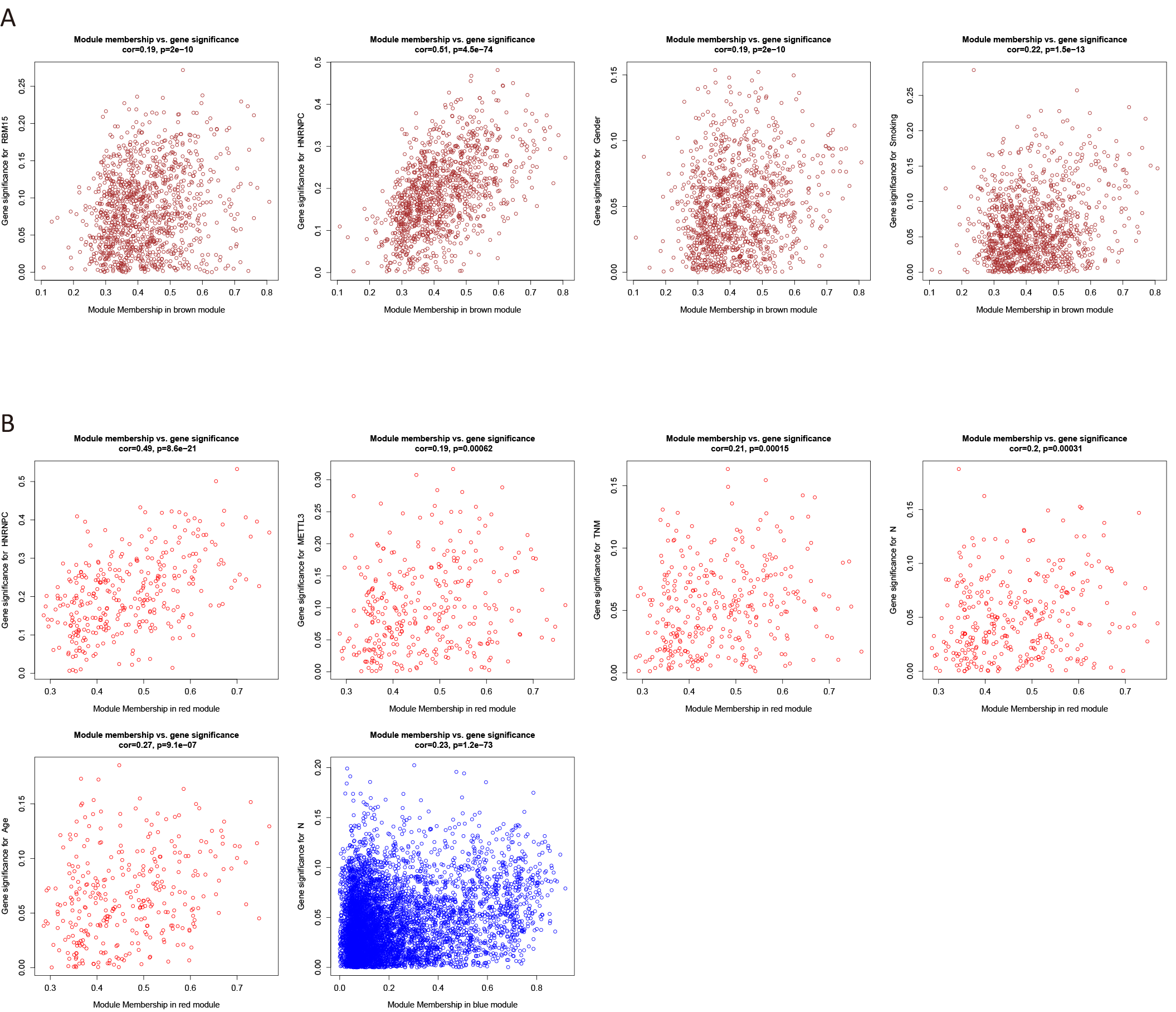

Supplement: Supplementary file 3 [file Image6.TIF]

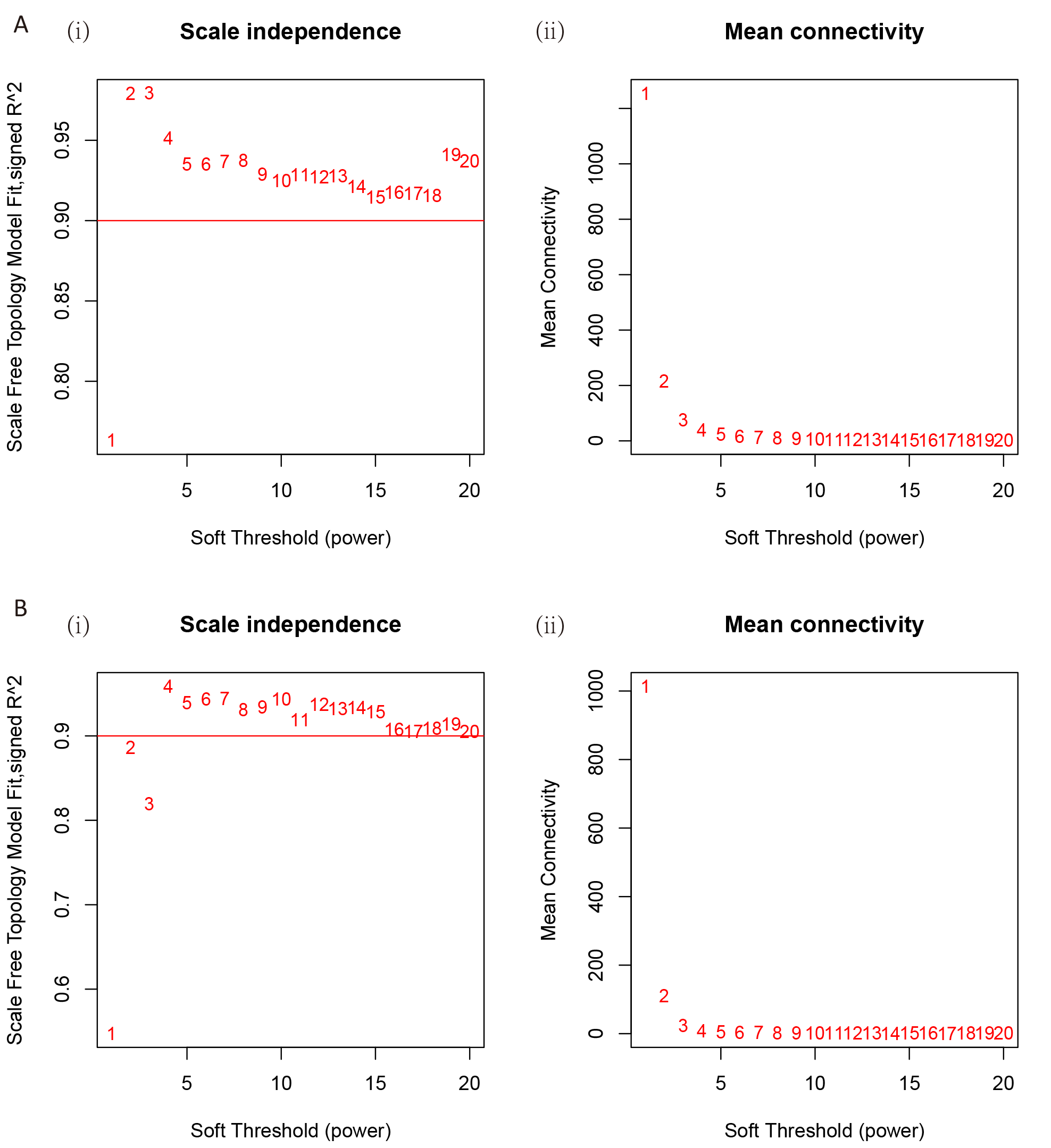

Supplement: Supplementary file 4 [file Image3.TIF]

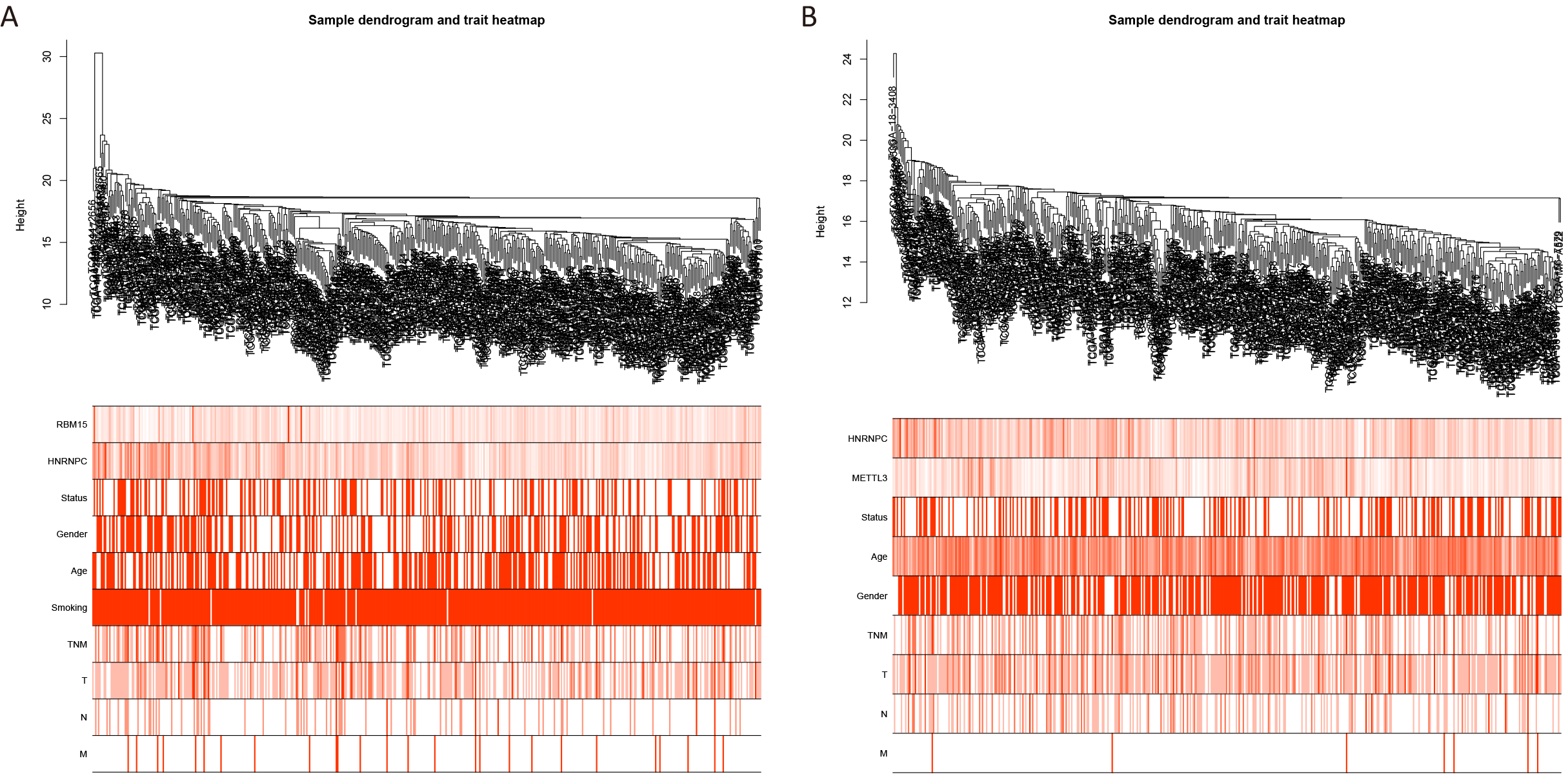

Supplement: Supplementary file 5 [file Image4.TIF]

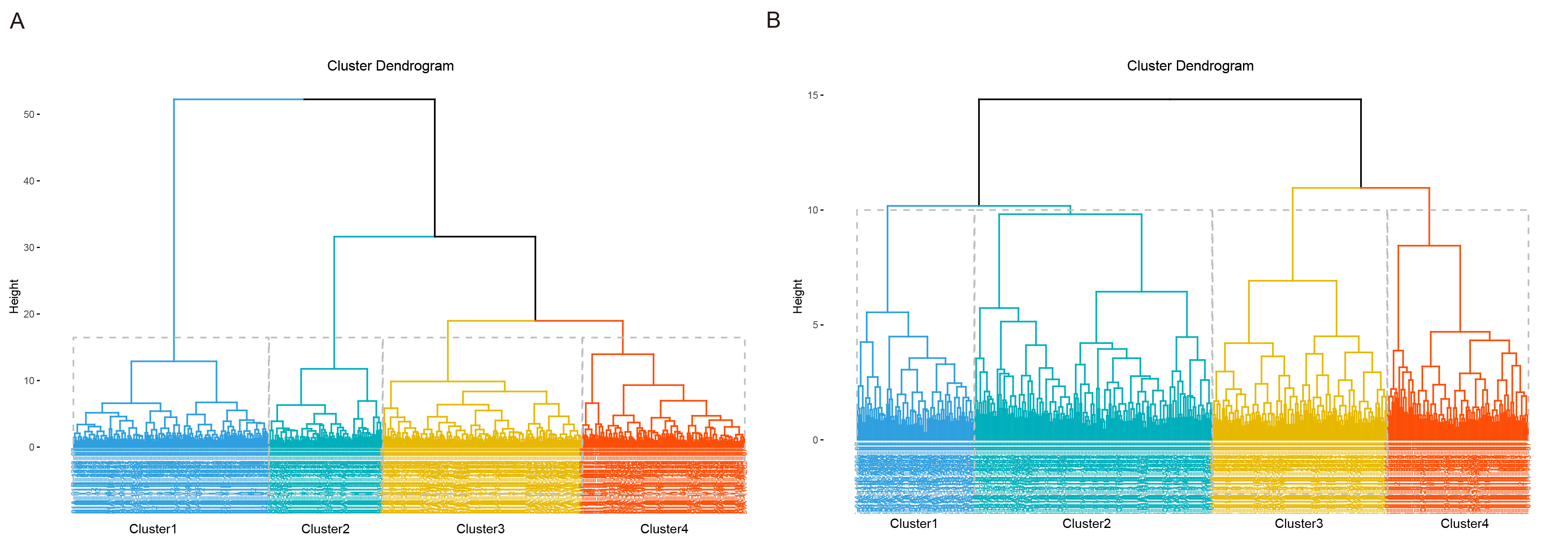

Supplement: Supplementary file 7 [file Image2.TIF]

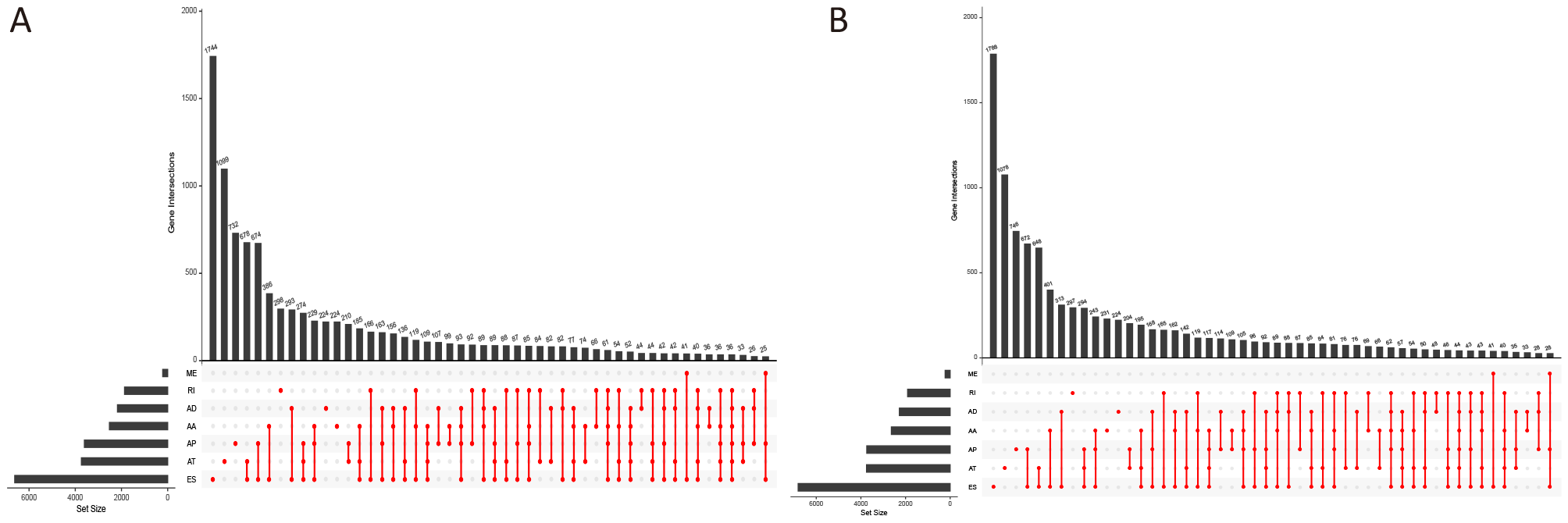

Supplement: Supplementary file 8 [file Image1.TIF]

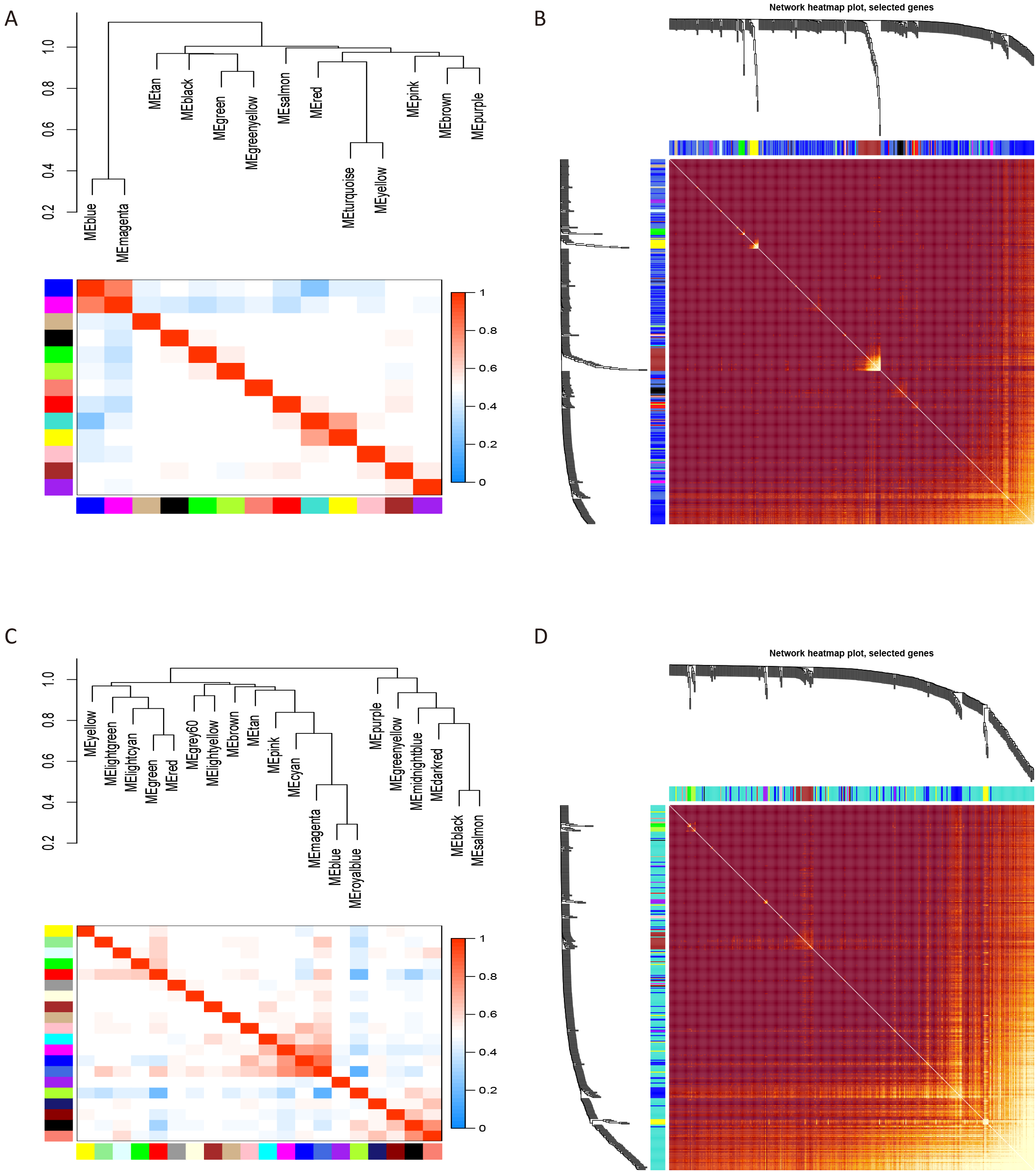

Supplement: Supplementary file 12 [file Image5.TIF]
